# Synthesis and Evaluation of Saccharide-Based Aliphatic and Aromatic Esters as Antimicrobial and Antibiofilm Agents †

**DOI:** 10.3390/ph12040186

**Published:** 2019-12-17

**Authors:** Raffaella Campana, Alessio Merli, Michele Verboni, Francesca Biondo, Gianfranco Favi, Andrea Duranti, Simone Lucarini

**Affiliations:** Department of Biomolecular Sciences, University of Urbino Carlo Bo, 61029 Urbino (PU), Italy; raffaella.campana@uniurb.it (R.C.); a.merli2@campus.uniurb.it (A.M.); m.verboni@campus.uniurb.it (M.V.); f.biondo1@campus.uniurb.it (F.B.); gianfranco.favi@uniurb.it (G.F.)

**Keywords:** sugar monoesters, biosurfactants, enzymatic synthesis, biocompatibility, antimicrobial agents, Minimum Inhibitory Concentration (MIC), antibiofilm agents, antifungal agents

## Abstract

A small library of sugar-based (i.e., glucose, mannose and lactose) monoesters containing hydrophobic aliphatic or aromatic tails were synthesized and tested. The antimicrobial activity of the compounds against a target panel of Gram-positive, Gram-negative and fungi was assessed. Based on this preliminary screening, the antibiofilm activity of the most promising molecules was evaluated at different development times of selected food-borne pathogens (*E. coli*, *L. monocytogenes*, *S. aureus*, *S. enteritidis*). The antibiofilm activity during biofilm formation resulted in the following: mannose C10 > lactose biphenylacetate > glucose C10 > lactose C10. Among them, mannose C10 and lactose biphenylacetate showed an inhibition for *E. coli* 97% and 92%, respectively. At MICs values, no toxicity was observed on Caco-2 cell line for all the examined compounds. Overall, based on these results, all the sugar-based monoesters showed an interesting profile as safe antimicrobial agents. In particular, mannose C10 and lactose biphenylacetate are the most promising as possible biocompatible and safe preservatives for pharmaceutical and food applications.

## 1. Introduction

Food pathogens are responsible for significant economic losses in the food industry and cause different human diseases [1]. In fact, bacteria and fungi can be toxic for humans, mainly due to the production of poisonous substances such as enterotoxins and mycotoxins, which can be found in contaminated food. Since some microorganisms and their toxins are thermostable, they cannot be destroyed by typical food conservation and preparation methods (cooling, freezing, cooking, frying, etc.) [2,3]. Indeed, recent studies by the Centers for Disease Control and Prevention estimated new cases of food-related illness in the United States at around 50 million, resulting in approximately 3000 deaths and 130,000 hospitalizations every year [4,5].

One of the bacterial growth modes is the development of the biofilm, which can be considered as a basic survival strategy in a wide range of environmental, industrial and clinical settings [6]. Biofilms are sessile communities of bacterial cells attached to each other and/or to surfaces or interfaces, which are embedded in a self-produced matrix of extracellular polymeric substances (EPS) that play an important role in protecting microorganisms against adverse environmental conditions, including the action of most antimicrobial agents [7].

Some of the common disinfection practices in the food industry, such as chemical-based [8] and irradiation [9], are very effective. However, they are frequently expensive, dangerous and risky for human health and the environment [10]. Moreover, some of these practices can remove biofilms but do not kill cells embedded into the deeper layer of biofilms, allowing them to later reattach to other surfaces and create a new biofilm [11]. Other practices, such as the use of common food preservatives (e.g., maleic acid or potassium sorbate), are now proving to be unsafe and toxic [12,13].

Many studies have evaluated the efficacy of natural compounds, extracted from bacterial cultures, or aromatic plants against food pathogens, as well as their potential to eradicate biofilms [14]. These compounds may exert a high level of lethal activity against pathogens, are efficient in penetrating the structure of the biofilm, and can be easily degraded in the environment [15]. In our opinion, there is still plenty of room for research into new natural or synthetic antimicrobial food additives with a safe toxicological profile.

In this context, sugar-based esters, a class of compounds constituted of a sugar moiety linked via an ester bond to aliphatic fatty acid chains, are amphiphilic, biodegradable, non-toxic, non-irritant, tasteless, odorless molecules, and are becoming widely used in food, pharmaceutical, and cosmetic industries [16,17,18].

Sugar-based ester surfactants have also the advantage of being synthesizable from sustainable and renewable resources (e.g., fatty acids and carbohydrates) by simple chemical esterification, and can be also produced by green methods, via enzymatic reactions or microbial fermentations. In the enzyme-catalyzed syntheses, the most utilized inductors are lipases, proteinases and glucosidases [19]. Lipase-catalyzed reactions are certainly the most studied, in order to determine the best solvent/solvent mixture, substrates ratio, temperatures, reaction times, type and concentration of the enzyme [20]. However, lipases are very selective, even in term of substrates. In fact, some lipophilic reactants, such as aromatic or polyunsaturated fatty acids, could not undergo the esterification process catalyzed by these enzymes. In these few cases, chemical esterification could be the main road to explore. Among all sugar-based surfactants, sucrose and glucose esters are the most studied and applied derivatives [21,22,23,24], while other derivatives, such as lactose esters, which can be easily obtained from waste whey, and mannose esters, have received less attention.

In this work, we report enzymatic and chemical synthetic procedures applied to obtain sugar-fatty acids esters, based on an esterification reaction between monosaccharide (glucose or mannose), or disaccharide (lactose) sugars and aliphatic (C8, C10, C12, C14 and C16 saturated fatty acids) or aromatic (phenylacetic, biphenylacetic, triphenylacetic and *p*-phenylbenzoic) acids. These compounds were tested in order to determine their minimum inhibitory concentration (MIC) values against different Gram-positive and Gram-negative bacteria, and fungi. Secondarily, the most promising compounds were also evaluated concerning their antibiofilm activity and biocompatibility profile. Notably, an extensive comparison of a large number of aliphatic and aromatic biocompatible sugar-based surfactants as antimicrobial and, mostly, as antibiofilm agents, is still lacking in the literature.

## 2. Results and Discussion

### 2.1. Chemistry

A small library of sugar-based amphiphilic esters from natural monosaccharides (α-d-mannose, α-d-glucose) or disaccharide (α-d-lactose) and saturated fatty acids [caprylic (C8), capric (C10), lauric (C12), myristic (C14), palmitic (C16)] or various aromatic acids (phenylacetic, *p*-phenylbenzoic, biphenylacetic, triphenylacetic) was designed and synthesized.

For all sugar-based surfactants, some physico-chemical properties, such as hydrophilic–lipophilic balance (HLB), octanol–water portion coefficient (logP) and topological polar surface area, (TPSA) were calculated (Table 1). In this context, the monosaccharide esters (entry 1–10) could be classified as lipophilic surfactants (HLB < 9) and have the potential to act as water-in-oil emulsifiers (5 < HLB < 8). Moreover, they have a TPSA lower than 140 Å^2^ (116.5), resulting in their being good at permeating peripheral cell membranes. On the other hand, lactose esters (entry 11–19) could be classified as hydrophilic surfactants (HLB > 10) and act as oil-in-water emulsifiers. The aromatic lactose esters (entry 16–19) generally show a lower logP than aliphatic ones (entry 11–15) with similar HLB, possibly having higher solubility in water. The aromatic lactose esters could be also very interesting because of their peculiar properties, possibly due to their different conformations in solution in comparison to aliphatic analogues.

**Scheme 1:**
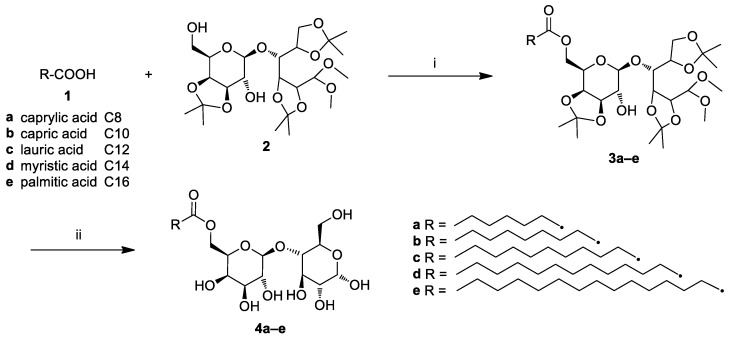
Reagents and conditions: (**i**) Lipozyme^®^, toluene, 75 °C, 12 h; (**ii**) HBF_4_/Et_2_O, CH_3_CN, 30 °C, 3 h.

The 6’-*O*-acyllactose aliphatic esters were synthesized by a two steps procedure (Scheme 1).

Firstly, the appropriate fatty acid (**1a–e**) was coupled with lactose tetra acetal (LTA) (**2**) using the catalyzing lipase Lipozyme^®^ in dry toluene furnished LTA esters **3a–e**. Subsequent deprotection of the latter by HBF_4_ led to a precipitate, which was filtered and recrystallized to obtain **4a–e**. Importantly, Lipozyme^®^ could be recovered and reused at least three times with no significant loss of activity, thereby reducing processing costs.

**Scheme 2:**
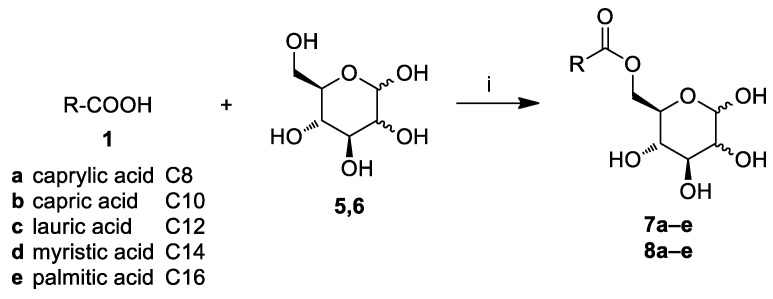
Reagents and conditions: (**i**) Novozyme, MS 4 Å, dry acetone, 96 h, rt.

Instead, enzymatic syntheses of 6-*O*-acylmannose and 6-*O*-acylglucose esters were conducted using a previously reported method [27] (Scheme 2).

In detail, the opportune aliphatic acid (**1a–e**) was coupled with α-D-mannose (**5**) or α-D-glucose (**6**) catalyzed by lipase Novozyme in dry acetone and molecular sieves to obtain the corresponding 6-*O*-acylmonosaccharide esters (**7a–e**, **8a–e**).

**Scheme 3:**
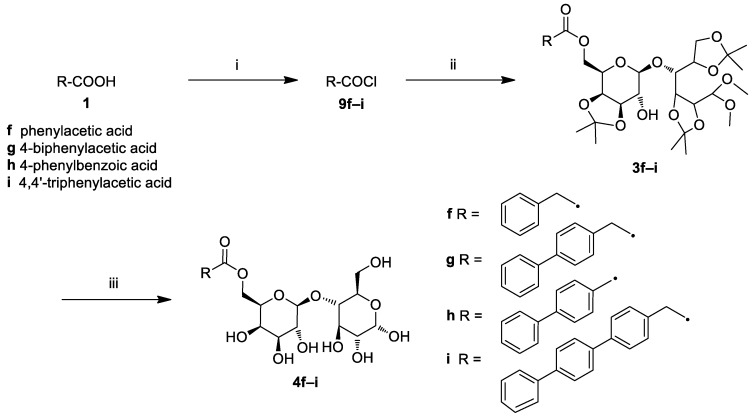
Reagents and conditions: (**i**) (COCl)_2_, dry DMF, rt, 2 h; (**ii**) **2**, DIPEA, dry CH_2_Cl_2_, 0 °C, 1 h then rt, 16 h; (**iii**) HBF_4_/Et_2_O, dry CH_3_CN, 30 °C, 3 h.

On the other hand, 6’-*O*-acyllactose aromatic esters (**4f–i**) were synthesized exploiting a conventional esterification method, since the Lipozyme^®^ (and other lipases) do not tolerate aromatic acid substrates (data not shown) (Scheme 3).

Regarding the starting materials (**1f–i**), three aromatic acids are commercially available (**1f–h**), while the triphenylacetic acid (**1i**) was synthesized following a reported protocol (see Supplementary Materials) [28]. With regards to acyl chloride activation, firstly the opportune aromatic acid (**1f–i**) was transformed into the corresponding acyl chloride by treatment with oxalyl chloride [(COCl)_2_] in the presence of a catalytic amount of dimethylformamide (DMF), and then it was coupled with LTA (**2**) in a 1:1 ratio and *N*,*N*-diisopropylethylamine (DIPEA) in dichlorometane (CH_2_Cl_2_) as a solvent to give the corresponding LTA monoesters (**3f–i**). Notably, neither 2’-*O*-acyllactose aromatic esters nor diester derivatives were detected from the crude mixture. The target esters (**4f–i**) were obtained as pure white solids by filtration, trituration and liofilization after precipitation under acidic deprotection conditions.

Finally, although some of the sugar-based esters here presented have already been published for other applications and also as possible antibacterial agents [22,23,24,29,30,31,32,33], a detailed comparative study concerning the properties of a relative large number of both aliphatic and aromatic amphiphilic carbohydrate esters as antimicrobial (Gram-positive, Gram-negative, and fungi) and mostly antibiofilm agents is still lacking.

### 2.2. Antimicrobial Activity

The antibacterial activity of sugar-based esters has evidenced variable results on different bacterial species. Indeed, in some studies the inhibition of Gram-negative bacteria was reported [34,35], while, in others, a better antimicrobial activity was shown on Gram-positive [36,37]. In the present work, sugar-based esters with different hydrophobic chains and polar heads displayed similar antibacterial activity against different tested strains, with no difference between Gram-positive and Gram-negative bacteria. In detail, MICs values of 256 µg/mL were determined for glucose C10 against Gram-positive (*E. faecalis* ATCC 29212, *L. monocytogenes* ATCC 7644, *S. aureus* ATCC 43387) and Gram-negative (*E. coli* O157:H7 ATCC 35150, *K. pneumoniae* ATCC 13833, *P. aeruginosa* ATCC 9027, *S. enteritidis* ATCC 13076) bacteria, as well as for *C. albicans* ATCC 10231. On the contrary, MICs were not determined (>256 µg/mL) for glucoses C12, C14 and C16, and, in some cases, for glucose C8 (*S. aureus* ATCC 43387, *K. pneumoniae* ATCC 13833 and *P. aeruginosa* ATCC 9027). Regarding the mannose series, the greatest antimicrobial activity (256 µg/mL) was observed for mannose C10 and C12 against all the pathogens, while in the case of mannoses C8, C14 and C16, MICs resulted in >256 µg/mL. A similar trend was shown for the lactose series that, in most cases, did not inhibit microbial growth at MICs lower than 256 µg/mL. Also, in this case, lactose C10 was the most active sugar-based aliphatic fatty acid ester, with a MIC of 128 µg/mL against *E. faecalis* ATCC 29212 and *C. albicans* ATCC 10231, and 256 µg/mL for all the other microorganisms. The last series, including lactose aromatic esters, showed MICs of 256 µg/mL for all the tested microorganisms and only in two cases the MICs were not determined (>256 µg/mL for *S. aureus* ATCC 43387 and *P. aeruginosa* ATCC 9027). It can be noted that, in most cases, the antibacterial activity of the examined sugar-based esters decreased rapidly with the increasing length of the fatty acid chain, as reported in the literature [38]. With regard to the internal control, gentamicin inhibited the growth of *S. enteritidis* ATCC 13076 with the lowest MIC value of 4 µg/mL, while the highest MIC (64 µg/mL) was observed for *E. faecalis* ATCC 29212 (Table 2). As a comparison, *C. albicans* ATCC 10231 was sensitive to fluconazole at MIC of 1 µg/mL, while the parabens mixture showed MIC values > 1024 µg/mL for all the examined microrganisms.

### 2.3. Inhibition of the Biofilm Formation at Different Times of Development

As known, microbial cells organized in biofilms are more resistant to antibiotics and some physical treatments [39,40,41]. For this reason, they represent a serious problem for infectious disease outbreaks. The process of biofilm formation is dynamic and involves at least four stages: planktonic, reversible and irreversible attachment, maturation, and dispersion [42]. In addition, in each stage the formation of various phenotypes is regulated by a different gene expression. One of the possible approaches to controlling the biofilm is to limit or inhibit its formation stage by searching for molecules endowed with antibiofilm activity, such as the sugar-based esters herein studied. However, it is reasonable that biofilms in different stages may have a different susceptibility to these molecules.

**Figure 1:**
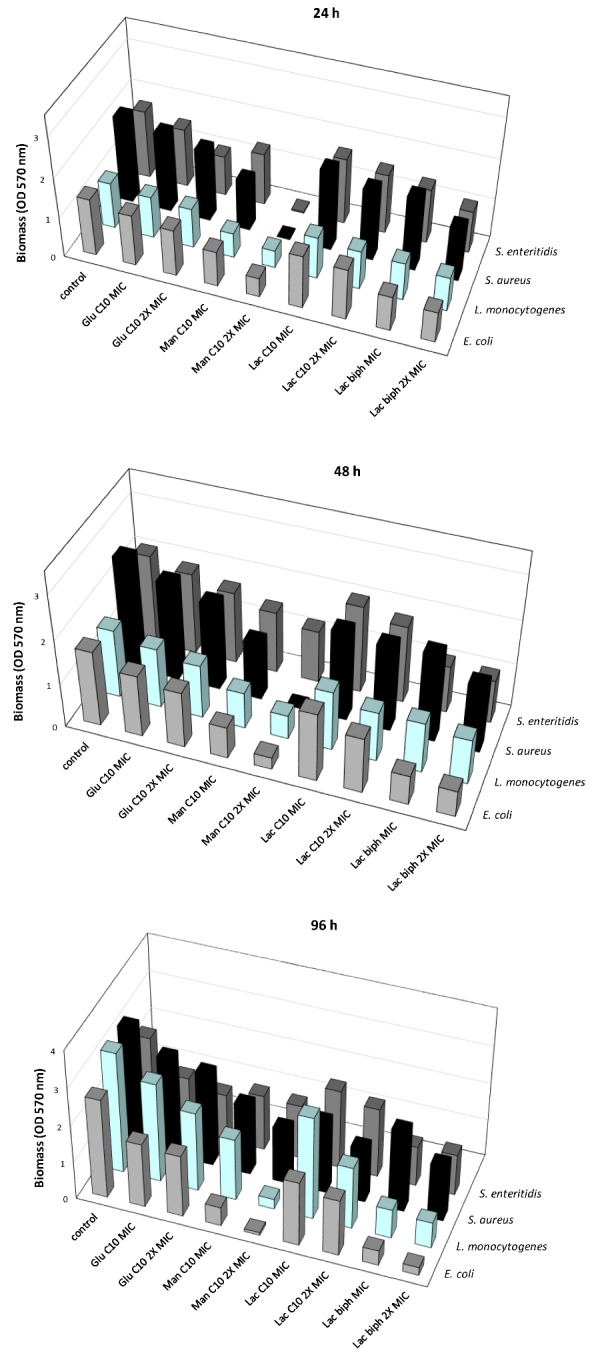
Effect of sugar-based esters at their MIC and 2× MIC values on the biofilm formation of different food-borne pathogens after 24 h, 48 h and 96 h of incubation.

On the basis of their assessed antimicrobial activity, four sugar-based esters were selected, three of which belong to the aliphatic series [i.e., Glucose C10 (Glu C10), Mannose C10 (Man C10), Lactose C10 (Lac C10)] and one to the aromatic series [i.e., lactose biphenylacetate (Lac biph)]. During the first 24 h of biofilm formation glucose C10, mannose C10, lactose C10 and lactose biphenylacetate at their MIC and 2× MIC values were able to differently reduce biofilm formation, generally in a dose-dependent way (Figure 1).

Mannose C10 was the most active in reducing the biofilm formation of all the examined microorganisms, with OD570 values lower than the corresponding untreated controls; in the case of *E. coli* O157:H7 ATCC 35150, OD values of 0.8789 and 0.4783 were registered in the presence of mannose at its MIC and 2× MIC values, respectively, compared to 1.424 of the control (*p* < 0.01); similarly, *L. monocytogenes* ATCC 7644 presented OD values of 0.6182 and 0.4328, with mannose at MIC and 2× MIC, respectively, in comparison to 1.149 of the control biofilm (*p* < 0.01). *S. aureus* ATCC 43387 and *S. enteritidis* ATCC 13076 biofilm formation was strongly inhibited, having the lowest OD values of 0.009 and 0.0225 observed for mannose at 2× MIC, compared to OD values of 2.138 and 1.699 of the control biofilms (*p* < 0.001). Regarding the other sugar fatty acids, a relevant biofilm reduction was observed with glucose C10 at 2× MIC values, particularly against *L. monocytogenes* ATCC 7644, compared to the control (OD 0.986 vs. 1.149) (*p* < 0.01), *S. aureus* ATCC 43387 (OD 1.775 vs 2.138) (*p* < 0.05), or to the untreated biofilm *S. enteritidis* ATCC 13076 (OD 0.998 vs. 1.699) (*p* < 0.01), while a moderate inhibition was observed in the case of *E. coli* O157:H7 ATCC 35150 compared to the related control (OD 1.158 vs. 1.424) (*p* < 0.05). A similar trend was observed with lactose C10, which was more active at a 2× MIC concentration, even with a similar OD value in comparison to that related to the untreated controls (*p* < 0.05). Interestingly, lactose biphenylacetate was able to reduce the biofilm formation of all the examined bacteria, with the lowest OD values of 0.856 and 0.755 at MIC and 2× MIC, respectively, compared to the OD value of 1.424 of the related control, in the case of *E. coli* O157:H7 ATCC 35150 (*p* < 0.05). The OD values that resulted were also always lower than those of the corresponding controls in the case of *L. monocytogenes* ATCC 7644 (OD 0.925 and 0.837 at MIC and 2× MIC, respectively), *S. aureus* ATCC 43387 (OD 1.801 and 1.304 at MIC and 2× MIC, respectively) and *S. enteritidis* ATCC 13076 (OD 1.349 and 1.047 at MIC and 2× MIC, respectively) (*p* < 0.05).

With a prolonged biofilm formation time of 48 h, the produced biomass increased for all the examined control biofilms, while the presence of sugar fatty acids, in most cases, affected the biomass production, thus indicating that these molecules still inhibit biofilm formation (Figure 1). As shown, this trend was particularly evident for mannose C10 and lactose biphenylacetate, while, in the case of glucose C10 and lactose C10, the effect was less remarkable, even if biomass production was lower than that of the related controls. Specifically, in the case of *S. aureus* ATCC 43387, a strong inhibition of biofilm formation was observed in the presence of mannose at 2× MIC concentration, with no detectable OD. In the 48 h biofilms of *E. coli* O157:H7 ATCC 35150, biomass production reached OD values of 0.714 (*p* < 0.05) and 0.267 (*p* < 0.01) with mannose C10 at MIC and 2× MIC, respectively, while an OD of 1.708 was obtained in the related control biofilm. Analogously, biomass production in *L. monocytogenes* ATCC 7644 biofilms developed in the presence of the same compound reached OD values of 0.820 and 0.536 (MIC and 2× MIC) compared to that of 1.552 of the untreated control (*p* < 0.05); also, the biofilm formation of *S. enteritidis* ATCC 13076 was affected by mannose C10 (MIC: 1.397, 2× MIC: 1.179) (*p* < 0.05; *p* < 0.01) in comparison to the control biofilm (OD 2.059). A good antibiofilm activity was still evident in the presence of lactose biphenylacetate at 2× MIC, that was able to significantly reduce the biomass production of *E. coli* O157:H7 ATCC 35150 and *L. monocytogenes* ATCC 7644, with OD values of 0.587 and 1.014, respectively (*p* < 0.05), compared to the higher biomass produced by the corresponding control samples (OD 1.708 and 1.552, respectively). A lesser antibiofilm activity was observed during biofilm formation with glucose C10 and lactose C10. Indeed, for all the examined microorganisms, the production of biomass in the presence of the sugar esters examined was slightly lower than that of the related controls, the lowest OD values being obtained with glucose C10 and lactose C10 at 2× MIC in the case of *L. monocytogenes* ATCC 7644 biofilms (OD 1.211 and 1.121, respectively, vs. control OD 1.552).

The prolonged time of biofilm formation, with up to 96 h of incubation, resulted in a great increase in biomass production by each tested microorganism in the control samples (Figure 1). The presence of sugar esters in the culture medium continued to affect the biofilms formation, as evidenced by the reduction in the biomass produced by microorganisms under the growth conditions. In detail, the most remarkable effect on biofilm formation was induced by mannose C10 at 2× MIC concentration against *E. coli* O157:H7 ATCC 35150 and *L. monocytogenes* ATCC 7644, with OD values of 0.073 and 0.265, respectively, compared to the related control biofilms (OD 2.663 and 3.240, respectively) (*p* < 0.01). However, *S. aureus* ATCC 43387 and *S. enteritidis* ATCC 13076 biofilms also showed a reduced biomass after 96 h of incubation with 2× MIC mannose C10, with OD values of 1.475 and 1.450, respectively, in comparison to OD values of 3.251 and 2.362 for the control ones (*p* < 0.01). Lactose biphenylacetate also resulted in a prolonged time of biofilm formation, and was able to reduce the biomass production of *E. coli* O157:H7 ATCC 35150 and *L. monocytogenes* ATCC 7644 at 2× MIC (OD 0.214 and 0.689, respectively) (*p* < 0.01; *p* < 0.05), in comparison to the control biofilms (OD 2.663 and 3.240, respectively). As observed for mannose C10, the induced biomass reduction in *S. aureus* ATCC 43387 and *S. enteritidis* ATCC 13076 biofilms was lower than observed for *E. coli* O157:H7 ATCC 35150 and *L. monocytogenes* ATCC 7644 (*p* < 0.05). A negligible effect was evidenced for glucose C10 and lactose C10 against the tested food-borne pathogens, with OD values quite similar to those of the control biofilms. Several factors may explain the observed increased resistance of mature biofilms to the tested molecules, such as those of 48 h and 96 h. Firstly, bacterial cells in mature biofilms are likely to be in the stationary growth phase and, therefore, less susceptible to antimicrobials. In addition, the dead cells in the outer layers of mature biofilms could be nutrients enhancing the growth of microbial cells in deeper layers [43]. Also, it could be considered that the high thickness or high amount of EPS that characterizes mature biofilms may limit the transport and diffusion of sugar fatty acids through biofilms.

### 2.4. Inhibition of the Biofilm Formation Induced by Each Examined Sugar-Based Ester

In Table 3, the percentage values of inhibition of the biofilm formation induced by each compound against the tested microorganisms are summarized, and a reduction between 30% and 50% was arbitrarily considered as an index of antibiofilm activity. The antibiofilm activity during biofilm formation was as follows: mannose C10 > lactose biphenylacetate > glucose C10 > lactose C10. The most active sugar fatty acid was mannose C10, with percentages of biofilm formation inhibition greater than 30% at MIC concentrations and 90% at 2× MIC. The antibiofilm effect resulted in increased percentages from 24 h up to 96 h, as observed for *E. coli* O157:H7 ATCC 35150 (66.7% and 97.2%, respectively) and *L. monocytogenes* ATCC 7644 (62.3 and 91.8%), while, in the case of *S. enteritidis* ATCC 13076, the highest percentages of biofilm reduction were observed after different incubation hours in the shorter formation time (98.5%, 24 h) and, for *S. aureus* ATCC 43387, in the intermediate one (99.9%, 48 h). Similarly, the effect of lactose biphenylacetate was evident over time (up to 96 h), with percentages of biofilm formation inhibition ranging from 53.3% to 92.0% (*S. enteritidis* ATCC 13076 and *E. coli* O157:H7 ATCC 35150, respectively). On the contrary, glucose C10 and lactose C10 were unable to limit biofilm formation after 24 h and 48 h (with the only exception being *S. enteritidis* ATCC 13076 by glucose C10 at 2× MIC) reaching percentages >30% (from 34.6% to 55.3%) in the long period of biofilm development. Interestingly, mannose C10 and lactose biphenylacetate proved to be the most active compounds (already at their MIC values) against biofilms at the different times of formation (up to 5 days). Based on analysis of the obtained results, it is possible to assert that biphenyl portion could be considered as bioisosteric in comparison to linear aliphatic chain ones. The obtained data hypothesize that the presence of the tested molecules in the culture medium limit the initial biofilm formation and continue to interfere with the following stages of biofilm maturation.

### 2.5. MTT Toxicity Assay

**Figure 2:**
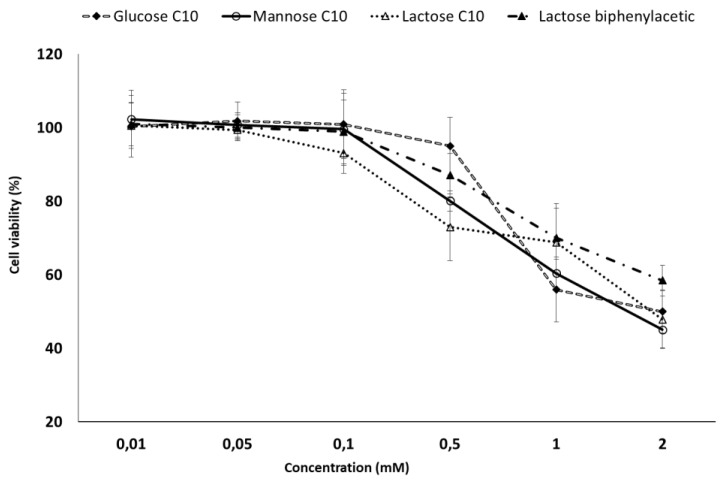
MTT assay of sugar-based surfactants on Caco-2 cell lines. The graph plots cell viability (%) as a function of different sugar fatty acids concentrations.

The cytotoxicity of the selected sugar fatty acids (glucose C10, mannose C10, lactose C10 and lactose biphenylacetate) was evaluated by 3-(4,5-dimethylthiazol-2-yl)-2,5-diphenyltetrazolium bromide (MTT) assay on Caco-2 cells (Figure 2). As known, the cytotoxic effect results from the interplay of different factors such as the ability of the hydrocarbon chain to insert into the lipid bilayer [44].

After 24 h exposition, all the compounds did not display toxicity to Caco-2 cells (cell viability ranging from 93.1 to 100%) at the low tested concentrations (0.01–0.1 mM), while a slight reduction in cell viability was observed in the case of lactose C10 0.5 mM (248 µg/mL, 73.0%). On the contrary, the highest concentrations (1 and 2 mM) were associated with a decrease in cell viability for all sugar fatty acids, with the lowest cell viability observed in the case of mannose C10 2 mM (669 µg/mL, 45.1%). Overall, it can be noted that, at MICs values, no considerable toxicity was observed for the selected sugar-based surfactants on the used cell line. This exploration can be considered a preliminary investigation on the toxicological profile of the examined surfactants, which should be confirmed by further studies.

## 3. Materials and Methods

### 3.1. Chemicals, Materials and Methods

Caprylic, capric, lauric, myristic and palmitic acids were purchased from TCI (Zwijndrecht, Belgium). Lactose monohydrate, α-d-glucose and α-D-mannose were purchased from Carlo Erba (Milan, Italy). Lipozyme^®^ (immobilized from Mucor miehei), Novozyme (lipase acrylic resin from *Candida antarctica*), *p*-toluenesulfonic acid, 2,2-dimethoxypropane, tetrafluoroboric acid diethyl ether complex [HBF_4_ Et_2_O], phenylacetic acid, biphenylacetic acid, *p*-phenylbenzoic acid, *p*-bromophenylbenzene, 4,4,4’,4’,5,5,5’,5’-octamethyl-2,2’-bis-1,3,2-dioxaborolane. [bis(pinacolato)diboron (B_2_pin_2_), [1,1’-bis(diphenylphosphino)ferrocene]dichloropalladium(II) [Pd(dppf)Cl_2_], 1,1’-bis(diphenylphosphino) ferrocene (dppf), tris(dibenzylideneacetone)dipalladium(0) [Pd_2_(dba)_3_], tricyclohexylphosphine [P(Cy)_3_], all organic solvents, gentamycin, fluconazole, penicillin, streptomycin, Dulbecco’s Modified Eagle Medium (DMEM), Fetal Calf Serum (FCS), non-essential amino acids and trypsin were purchased from Sigma-Aldrich (Milan, Italy). Prior to use, acetonitrile and acetone were dried with molecular sieves with an effective pore diameter of 4 Å and toluene was saturated with water. The structures of compounds were unambiguously assessed by MS, ^1^H NMR, ^13^C NMR, and IR. ESI-MS spectra were recorded with a Waters Micromass ZQ spectrometer in a negative or positive mode using a nebulizing nitrogen gas at 400 L/min and a temperature of 250 °C, cone flow 40 mL/min, capillary 3.5 kV and cone voltage 60 V; only molecular ions [M-H]^−^ or [M + NH_4_]^+^ or [M + Na]^+^ are given. ^1^H NMR and ^13^C NMR spectra were recorded on a Bruker AC 400 or 101, respectively, spectrometer and analyzed using the TopSpin software package. Chemical shifts were measured by using the central peak of the solvent. Column chromatography purifications were performed under “flash” conditions using Merck 230–400 mesh silica gel. TLC was carried out on Merck silica gel 60 F254 plates, which were visualized by exposure to ultraviolet light and by exposure to an aqueous solution of ceric ammonium molybdate.

### 3.2. General Procedure for the Synthesis of Lactose Fatty Acid Ester Derivatives *(**3a–e**)*

Lipozyme^®^ (0.078 g) was added to a solution of the opportune fatty acid (**1a–e**) (0.79 mmol) and 4-*O*-(3’,4’-*O*-isopropylidene-α-d-galactopyranosyl)-2,3:5,6-di-*O*-isopropylidene-1,1-di-*O*-methyl-D-glucopyranose (lactose tetra acetal, LTA) [45] (**2**) (0.402 g, 0.79 mmol) in dry toluene (0.5 mL) at 30 °C [46,47]. The mixture was stirred at 75 °C for 12 h, cooled and diluted with acetone, then the enzyme was filtered and the filtrate was concentrated. Purification of the residue by column chromatography (cyclohexane/EtOAc 8:2) gave **3a–e** as pale yellow oils. (Scheme 1)

6’-*O*-Octanoyl-4-*O*-(3’,4’-*O*-isopropylidene-β-d-galactopyranosyl)-2,3:5,6-di-*O*-isopropylidene-1,1-di-*O*-methyl-d-glucopyranose (**3a**) [47]

Yield = 55%. ^1^H NMR (400 MHz, CDCl_3_): δ = 0.92 (t, 3H, *J* = 6.7 Hz, CH_3_), 1.31 (s, 6H, 2 CH_3_), 1.33–1.37 (m, 18H), 1.39 (s, 3H, CH_3_), 1.41 (s, 3H, CH_3_), 1.44 (s, 3H, CH_3_), 1.49 (s, 3H, CH_3_), 1.61–1.67 (m, 2H, *CH*_2_CH_2_CO), 2.40 (t, 2H, *J* = 7.0 Hz, *CH*_2_CO), 3.45–3.47 (m, 6H, OCH_3_), 3.47 (dd, 1H, *J*_H2’-H3’_ = 7.1 Hz, *J*_H2’-H1’_ = 8.0 Hz, H^2’^), 3.91 (dd, 1H, *J*_H4-H3_ = 1.0 Hz, *J*_H4-H5_ = 5.0 Hz, H^4^), 4.04 (ddd, 1H, *J*_H5’-H6a’_ = 1.0 Hz, *J*_H5’-H4’_ = 2.0 Hz, *J*_H5’-H6b’_ = 7.0 Hz, H^5’^), 4.05 (dd, 1H, *J*_H6b-H5_ = 6.0 Hz, *J*_H6b-H6a_ = 8.5 Hz, H^6b^),4.08 (dd, 1H, *J*_H3’-H4’_ = 5.5 Hz, *J*_H3’-H2’_ = 7.0 Hz, H^3’^), 4.15 (dd, 1H, *J*_H3-H4_ = 1.0 Hz, *J*_H3-H2_ = 7.5 Hz, H^3^), 4.17 (dd, 1H, *J*_H6a-H5_ = 6.0 Hz, *J*_H6a-H6b_ = 8.5 Hz, H^6a^), 4.22 (dd, 1H, *J*_H4’-H5’_ = 2.0 Hz, *J*_H4’-H3’_ = 5.5 Hz, H^4’^), 4.27 (dd, 1H, *J*_H6b’-H5’_ = 7.0 Hz, *J*_H6b’-H6a’_ = 11.5 Hz, H^6b’^), 4.30 (dd, 1H, *J*_H6a’-H5’_ = 1.0 Hz, *J*_H6a’-H6b’_ = 11.5 Hz, H^6a’^), 4.30 (ddd, *J*_H5-H4_ = 5.0 Hz, *J*_H5-H6a_ ≅ *J*_H5-H6b_ = 6.0 Hz, H^5^), 4.41 (d, 1H, *J*_H1-H2_ = 6.0 Hz, H^1^), 4.51 (d, 1H, *J*_H1’-H2’_ = 8.0 Hz, H^1’^), 4.51 (dd, 1H, *J*_H2-H1_ = 6.0 Hz, *J*_H2-H3_ = 7.5 Hz, H^2^) ppm. ^13^C NMR (100 MHz, CDCl_3_): δ = 13.0, 22.3, 24.2, 24.6, 25.1, 25.5, 25.6, 26.2, 27.0, 28.8, 29.0, 29.1, 29.2, 29.3, 29.3, 29.4, 31.7, 33.5, 53.0, 55.1, 63.1, 65.5, 70.8, 73.3, 73.6, 75.4, 76.4, 76.8, 77.6, 79.4, 103.1, 105.7, 108.5, 109.7, 109.9, 173.8 ppm.

Compounds **3b–e** were previously characterized [48].

Compounds **3a–e** (0.25 mmol) were dissolved in [HBF_4_.(Et)_2_O]/H_2_O/dry CH_3_CN (2.1 mL, 1:5:500) and the mixture was stirred at 30 °C for 3 h [36,37]. The white solids precipitated were then filtered, washed with CH_3_CN, and dried (Na_2_SO_4_). Purification by recrystallization from methanol gave **4a–e** as white solids.

6’-*O*-Octanoyl-4-*O*-(β-d-galactopyranosyl)-d-glucopyranose (lactose caprylate, **URB1415**) (**4a**) [47]

Yield = 46%. MS (ESI): 467 [M–H]^−^, 486 [M + NH_4_]^+^, 491 [M + Na]^+^. ^1^H NMR (400 MHz, DMSO): δ = 0.87 (t, 3H, *J* = 6.5 Hz, CH_3_), 1.20–1.34 [m, 8H, (–CH_2_–)_n_], 1.52–1.55 (m, 2H, *CH*_2_CH_2_COOR), 2.31 (t, 2H, *J* = 7.5 Hz, *CH*_2_COOR), 3.17 (ddd, 1H, *J*_H2-H1_ = 4.0 Hz, *J*_H2-OH2_ = 7.0 Hz, *J*_H2-H3_ = 9.5 Hz, H^2^), 3.28 (dd, 1H, *J*_H4-H3_ ≅ *J*_H4-H5_ = 9.0 Hz, H^4^), 3.32–3.38 (m, 2H, H^2’^, H^3’^), 3.57 (dd, 1H, *J*_H3-H2_ ≅ *J*_H3-H4_ = 9.0 Hz, H^3^), 3.63–3.65 (m, 3H, H^6a^, H^6b^, H^4’^), 3.69–3.75 (m, 2H, H^5^, H^5’^), 4.09 (dd, 1H, *J*_H6a’-H5’_ = 4.0 Hz, *J*_H6a’-H6b’_ = 11.5 Hz, H^6a’^), 4.17 (dd, 1H, *J*_H6b’-H5’_ = 8.5 Hz, *J*_H6b’-H6a’_ = 11.5 Hz, H^6b’^), 4.20–4.24 (m, 2H, H^1’^, OH^3^), 4.43 (dd, 1H, *J*_OH6-H6a_ ≅ *J*_OH6-H6b_ = 6.0 Hz, OH^6^), 4.56 (d, 1H, *J*_OH2-H2_ = 7.0 Hz, OH^2^), 4.79 (d, 1H, *J*_OH4’-H4’_ = 5.0 Hz, OH^4’^), 4.86 (brs, 1H, OH), 4.90 (dd, 1H, *J*_H1-OH1_ = 4.5 Hz, *J*_H1-H2_ = 4.0 Hz, H^1^), 5.15 (brs, 1H, OH), 6.34 (d, 1H, *J*_OH1-H1_ = 4.5 Hz, OH^1^) ppm. ^13^C NMR (100 MHz, DMSO): δ = 14.4, 22.5, 24.8, 28.8, 28.9, 31.6, 33.8, 60.9, 63.8, 68.7, 70.2, 70.8, 71.7, 72.7, 72.9, 73.3, 81.5, 92.5, 104.0, 173.4 ppm.

Compounds **4b**, **4c**, **4d** and **4e** (**URB1379**, **URB1380**, **URB1381** and **URB1382**, respectively) were previously described [48].

### 3.3. General Procedure for the Synthesis of Glucose and Mannose Fatty Acid Ester Derivatives *(**7a–e**, **8a–e**)*

Novozyme 435 (Novozyme) (0.200 g) and molecular sieves 4 Å (0.400 g) were added to a solution of the opportune fatty acid (**1a–e**) (2.1 mmol) and α-d-mannose (**5**) or α-d-glucose (**6**) (0.126 g, 0.7 mmol) in dry acetone (4.2 mL) at room temperature [27]. The mixture was stirred at room temperature for 96 h, filtered, and the filtrate was concentrated. Purification of the residue by flash chromatography (EtOAc/cyclohexane 8:2) gave **7a–e** as white spongy solids and **8a–e** as white crystal solids. (Scheme 2).

6-*O*-Octanoyl-d-mannopyranose (mannose caprylate, **URB1389**) (**7a**) [29]

Yield = 57%, α/β = 1:0.5. ^1^H NMR (400 MHz, DMSO): δ = 0.86 (t, 3H+1.5H, *J* = 6.5 Hz, CH_3_), 1.24–1.26 (m, 8H+4H), 1.50–1.53 (m, 2H+1H, OCCH_2_*CH*_2_), 2.26–2.31 (m, 2H+1H, OC*CH*_2_CH_2_), 3.21–3.33 (m, 1.5H, H^3^^β^, H^4^^β^, H^5^^β^), 3.35–3.41 (m, 1H, H^4^^α^), 3.49–3.55 (m, 2H+0.5H, H^2^^α^, H^3^^α^, H^2^^β^), 3.70 (ddd, 1H, *J*_H5__α-H6b__α_ = 1.5 Hz, *J*_H5__α-H6a__α_ = 7.0 Hz, *J*_H5__α-H4__α_ = 9.0 Hz, H^5^^α^), 3.94–4.02 (m, 1H+0.5H, H^6a^^α^, H^6a^^β^), 4.27–4.32 (m, 1H+0.5H, H^6b^^α^, H^6b^^β^), 4.53–4.59 (m, 1H+1H, OH^2^^α^, H^1^^β^, OH^2^^β^), 4.63 (d, 1H, *J*_OH3__α-H3__α_ = 4.0 Hz, OH^3^^α^), 4.66 (d, 0.5H, *J*_OH3__β-H3__β_ = 5.5 Hz, OH^3^^β^), 4.86 (dd, 1H, *J*_H1__α-H2__α_ ≅ *J*_H1__α-OH1__α_ = 4.5 Hz, H^1^^α^), 4.89 (d, 1H, *J*_OH4__α-H4__α_ = 5.5 Hz, OH^4^^α^), 4.94 (d, 0.5H, *J*_OH4__β-H4__β_ = 5.0 Hz, OH^4^^β^), 6.26 (d, 0.5H, *J*_OH1__β-H1__β_ = 8.5 Hz, OH^1^^β^), 6.38 (d, 1H, *J*_OH1__α-H1__α_ = 4.5 Hz, OH^1^^α^) ppm. ^13^C NMR (100 MHz, DMSO): δ = 14.4 (1.5C), 22.5 (1.5C), 24.9 (1.5C), 28.8 (1.5C), 28.9 (1.5C), 31.6 (1.5C), 33.87 (0.5C), 33.93 (1C), 64.3 (C6, 0.5C), 64.7 (C6, 1C), 67.2 (C5, 0.5C), 67.6 (C5, 1C), 70.8 (C4, 1C), 70.9 (C3, 1C), 71.8 (C2, 1C), 72.0 (C2, 0.5C), 73.9 (C4, 0.5), 74.5 (C3, 0.5C), 94.5 (C1, C), 94.6 (C1, 0.5C), 173.4 (CO, 1.5C) ppm.

Compounds **7b–e** are described in the Supplementary Materials.

6-*O*-Octanoyl-d-glucopyranose (glucose caprylate, **URB1388**) (**8a**) [49]

Yield = 71%. ^1^H NMR (400 MHz, DMSO): δ = 0.86 (t, 3H, *J* = 7.0 Hz, CH_3_), 1.24–1.26 (m, 8H), 1.49–1.53 (m, 2H, OCCH_2_*CH*_2_), 2.28 (t, 2H, *J* = 7.5 Hz, OC*CH*_2_CH_2_), 3.04 (ddd, 1H, *J*_H4-OH4_ = 6.0 Hz, *J*_H4-H3_ = 9.0 Hz, *J*_H4-H5_ = 9.5 Hz, H^4^), 3.12 (ddd, 1H, *J*_H2-H1_ = 3.5 Hz, *J*_H2-OH2_ = 6.5 Hz, *J*_H2-H3_ = 9.0 Hz, H^2^), 3.43 (ddd, 1H, *J*_H3-OH3_ = 5.0 Hz, *J*_H3-H2_ ≅ *J*_H3-H4_ = 9.0 Hz, H^3^), 3.77 (ddd, 1H, *J*_H5-H6b_ = 2.0 Hz, *J*_H5-H6a_ = 6.0 Hz, *J*_H5-H4_ = 9.5 Hz, H^5^), 3.99 (dd, 1H, *J*_H6a-H5_ = 6.0 Hz, *J*_H6a-H6b_ = 12.0 Hz, H^6a^), 4.27 (dd, 1H, *J*_H6b-H5_ = 2.0 Hz, *J*_H6b-H6a_ = 12.0 Hz, H^6b^), 4.55 (d, 1H, *J*_OH2-H2_ = 6.5 Hz, OH^2^), 4.77 (d, H, *J*_OH3-H3_ = 5.0 Hz, OH^3^), 4.90 (dd, 1H, *J*_H1-H2_ = 3.5 Hz, *J*_H1-OH1_ = 4.5 Hz, H^1^), 5.05 (d, 1H, *J*_OH4-H4_ = 6.0 Hz, OH^4^), 6.35 (d, 1H, *J*_OH1-H1_ = 4.5 Hz, OH^1^) ppm. ^13^C NMR (100 MHz, DMSO): δ = 14.4, 22.5, 24.9, 28.8, 28.9, 31.6, 33.9, 64.3 (C6), 69.6 (C5), 71.0 (C4), 72.7 (C2), 73.3 (C3), 92.8 (C1), 173.4 (CO) ppm.

Compounds **8b–e** are described in the Supplementary Materials.

### 3.4. General Procedure for the Synthesis of Lactose Aromatic Fatty Acid Ester Derivatives *(**4f–i**)*

Oxalyl chloride (1.630 g, 1.08 mL, 12.80 mmol) and catalytic dry DMF (two drops) were added to the opportune acid (**1f–i**) (0.8 mmol). The mixture was stirred at room temperature for 2 h, diluted with CH_2_Cl_2_, and concentrated to give the desired acid chloride (**9f–i**) as pale yellow oils. The opportune **9f–i** in dry CH_2_Cl_2_ (2.4 mL) was then added dropwise at 0 °C to a solution of DIPEA (0.382 g, 0.51 mL, 2.96 mmol) and LTA (**2**) (0.300 g, 0.59 mmol) in dry CH_2_Cl_2_ (2.4 mL). The mixture was stirred at 0 °C for 1 h and at room temperature for 16 h, then extracted with CH_2_Cl_2_. The combined organic layers were washed with NaHCO_3_ saturated solution, dried (Na_2_SO_4_), filtered, and concentrated. Purification of the residue by column chromatography (cyclohexane/ethyl acetate 8:2) gave **3f–i** as pale yellow oils (Scheme 3).

6’-*O*-(2-Phenylethanoyl)-4-*O*-(3’,4’-*O*-isopropylidene-β-d-galactopyranosyl)-2,3:5,6-di-*O*-isopropylidene-1,1-di-*O*-methyl-d-glucopyranose (**3f**)

Yield = 83%. ^1^H NMR (400 MHz, CDCl_3_): δ = 1.22 (s, 3H, CH_3_), 1.27 (s, 3H, CH_3_), 1.32 (s, 3H, CH_3_), 1.33 (s, 3H, CH_3_), 1.43 (s, 6H, 2CH_3_), 3.360 (s, 3H, OCH_3_), 3.364 (s, 3H, OCH_3_), 3.48 (dd, 1H, *J*_2’-3’_ = 7.0 Hz, *J*_2’-1’_ = 8.0 Hz, H^2’^), 3.59 (s, 2H, CH_2_), 3.84 (dd, 1H, *J*_3-4_ = 2.0 Hz, *J_3-2_* = 7.6 Hz, H^3^), 3.86 (ddd, 1H, *J*_5-6a_ = 2.0 Hz, *J*_5-6b_ ≅ *J*_5-4_ = 6.0 Hz, H^5^), 3.92–3.95 (m, 2H), 3.97 (dd, 1H, *J*_3’-4’_ = 5.6 Hz, *J*_2’-3’_ = 7.0 Hz, H^3’^), 4.00 (dd, 1H, *J*_1_ ≅ *J*_2_ = 1.5 Hz), 4.09 (dd, 1H, *J_1_* = 6.4 Hz, *J*_2_ = 8.8 Hz), 4.22 (ddd, 1H, *J*_5’-6’a_ = 2.4 Hz, *J*_5’-4’_ ≅ *J*_5’-6’b_ = 6.5 Hz, H^5’^), 4.23–4.26 (m, 1H), 4.27 (dd, 1H, *J*_4-3_ = 2.0 Hz, *J*_4-5_ = 6.0 Hz, H^4^), 4.30 (d, 1H, *J*_1-2_ = 6.0 Hz, H^1^), 4.34 (d, 1H, *J*_1’-2’_ = 8.0 Hz, H^1’^), 4.39 (dd, 1H, *J*_2-1_ = 6.0 Hz, *J*_2-3_ = 7.6 Hz, H^2^), 7.19–7.26 (m, 5H, ArH) ppm. ^13^C NMR (100 MHz, CDCl_3_): δ = 24.4, 25.7, 26.2, 26.4, 27.3, 28.1, 41.1, 53.5, 56.2, 63.4, 64.6, 71.2, 73.1, 74.1, 75.2, 76.4, 77.8, 78.0, 78.9, 103.7, 105.3, 108.3, 110.26, 110.30, 127.2, 128.6, 129.2, 133.8, 171.3 ppm.

6’-*O*-[2-(4-Phenyl)phenylethanoyl]-4-*O*-(3’,4’-*O*-isopropylidene-β-d-galactopyranosyl)-2,3:5,6-di-*O*-isopropylidene-1,1-di-*O*-methyl-d-glucopyranose (**3g**)

Yield = 44%. MS (ESI): 720 [M + NH_4_]^+^, 725 [M + Na]^+^, 701 [M–H]^−^. ^1^H NMR (400 MHz, CDCl_3_): δ = 1.16 (s, 3H, CH_3_), 1.26 (s, 3H, CH_3_), 1.29 (s, 3H, CH_3_), 1.31 (s, 3H, CH_3_), 1.42 (s, 6H, 2 CH_3_), 3.30 (s, 3H, OCH_3_), 3.33 (s, 3H, OCH_3_), 3.46 (dd, 1H, *J*_2’-3’_ = 7.0 Hz, *J*_2’-1’_ = 8.0 Hz, H^2’^), 3.82–3.87 (m, 3H), 3.91–3.95 (m, 2H), 3.97 (dd, 1H, *J*_1_ ≅ *J*_2_ = 1.5 Hz), 4.09 (dd, 1H, *J*_1_ = 6.4 Hz, *J*_2_ = 8.8 Hz), 4.22 (ddd, 1H, *J*_5’-6’a_ = 2.4 Hz, *J*_5’-4’_ ≅ *J*_5’-6’b_ = 6.8 Hz, H^5’^), 4.23–4.26 (m, 1H), 4.27 (dd, 1H, *J*_4-3_ = 2.0 Hz, *J*_4-5_ = 6.0 Hz, H^4^), 4.30 (d, 1H, *J*_1-2_ = 6.0 Hz, H^1^), 4.34 (d, 1H, *J*_1’-2’_ = 8.0 Hz, H^1’^), 4.38 (dd, 1H, *J*_2-1_ = 6.0 Hz, *J*_2-3_ = 7.6 Hz, H^2^), 4.99 (s, 2H, CH_2_), 7.15–7.27 (m, 9H, ArH) ppm. ^13^C NMR (100 MHz, DMSO): δ = 26.0, 26.5, 27.0, 27.1, 27.8, 28.4, 53.9, 55.7, 56.1, 63.8, 66.1, 69.9, 72.7, 73.2, 75.3, 76.3, 77.4, 77.5, 79.5, 103.7, 105.5, 108.5, 109.2, 109.6, 127.6, 128.7, 128.9, 129.0, 139.2, 139.4, 172.2 ppm.

6’-*O*-[2-(4-Phenyl)benzoyl]-4-*O*-(3’,4’-*O*-isopropylidene-β-d-galactopyranosyl)-2,3:5,6-di-*O*-isopropylidene-1,1-di-*O*-methyl-d-glucopyranose (**3h**)

Yield = 8%. MS (ESI): 706 [M + NH_4_]^+^, 711 [M + Na]^+^. ^1^H NMR (400 MHz, CDCl_3_): δ = 1.26 (s, 3H, CH_3_), 1.28 (s, 3H, CH_3_), 1.30 (s, 3H, CH_3_), 1.32 (s, 3H, CH_3_), 1.43 (s, 3H, CH_3_), 1.46 (s, 3H, CH_3_), 3.27 (s, 3H, OCH_3_), 3.28 (s, 3H, OCH_3_), 3.54 (dd, 1H, *J*_2’-3’_ = 7.0 Hz, *J*_2’-1’_ = 8.0 Hz, H^2’^), 3.84 (dd, 1H, *J*_3-4_ = 2.0 Hz, *J*_3-2_ = 7.6 Hz, H^3^), 3.94 (dd, 1H, *J*_1_ = 6.8 Hz, *J*_2_ = 8.8 Hz), 4.02–4.08 (m, 3H), 4.10 (dd, 1H, *J*_1_ = 6.8 Hz, *J*_2_ = 8.8 Hz), 4.15 (dd, 1H, *J*_4-3_ = 2.0 Hz, *J*_4-5_ = 6.0 Hz, H^4^), 4.19–4.23 (m, 1H), 4.22 (d, 1H, *J*_1-2_ = 5.6 Hz, H^1^), 4.41 (d, 1H, *J*_1’-2’_ = 8.0 Hz, H^1’^), 4.41 (dd, 1H, *J*_2-1_ = 5.6 Hz, *J*_2-3_ = 7.6 Hz, H^2^), 4.49 (dd, 1H, *J*_6b’-5’_ = 7.2 Hz, *J*_6b’-6a’_ = 11.6 Hz, H^6b’^), 4.56 (dd, 1H, *J*_6a’-5’_ = 4.8 Hz, *J*_6a’-6b’_ = 11.6 Hz, H^6a’^), 7.30–7.33 (m, 1H, ArH), 7.37–7.41 (m, 2H, ArH), 7.53–7.55 (m, 2H, ArH), 7.59 (d, 2H, *J* = 8.5 Hz, ArH), 8.05 (d, 2H, *J* = 8.5 Hz, ArH) ppm.

6’-*O*-[2-(4,4’-Biphenyl)phenylethanoyl]-4-*O*-(3’,4’-*O*-isopropylidene-β-d-galactopyranosyl)-2,3:5,6-di-*O*-isopropylidene-1,1-di-*O*-methyl-d-glucopyranose (**3i**)

Yield = 17%. MS (ESI): 796 [M + NH_4_]^+^, 801 [M + Na]^+^, 777 [M–H]^−^. ^1^H NMR (400 MHz, CDCl_3_): δ = 1.22 (s, 3H, CH_3_), 1.26 (s, 3H, CH_3_), 1.32 (s, 3H, CH_3_), 1.34 (s, 3H, CH_3_), 1.42 (s, 3H, CH_3_), 1.43 (s, 3H, CH_3_), 3.37 (s, 3H, OCH_3_), 3.38 (s, 3H, OCH_3_), 3.47–3.51 (m, 1H, H^2’^), 3.65 (s, 2H, CH_2_), 3.84 (dd, 1H, *J*_3-4_ = 2.0 Hz, *J*_3-2_ = 7.6 Hz, H^3^), 3.89 (ddd, 1H, *J*_5-6a_ = 2.0 Hz, *J*_5-6b_ ≅ *J*_5-4_ = 6.0 Hz, H^5^), 3.94 (dd, 1H, *J*_4’-5’_ = 6.8 Hz, *J*_3’-4’_ = 8.5 Hz, H^4’^), 3.96–3.99 (m, 3H), 4.10 (dd, 1H, *J*_3’-2’_ = 6.4 Hz, *J*_3’-4’_ = 8.5 Hz, H^3’^), 4.22 (ddd, 1H, *J*_5’-6’a_ = 2.0 Hz, *J*_5’-4’_ ≅ *J*_5’-6’b_ = 6.5 Hz, H^5’^), 4.25–4.33 (m, 2H), 4.31 (d, 1H, *J*_1-2_ = 6.4 Hz, H^1^), 4.36 (d, 1H, *J*_1’-2’_ = 8.0 Hz, H^1’^), 4.41 (dd, 1H, *J*_2-1_ = 6.4 Hz, *J*_2-3_ = 7.6 Hz, H^2^), 7.27–7.32 (m, 3H, ArH), 7.36–7.41 (m, 2H, ArH), 7.54 (d, 2H, *J* = 8.5 Hz, ArH), 7.57 (d, 2H, *J* = 8.5 Hz, ArH), 7.57–7.59 (m, 4H, ArH) ppm. ^13^C NMR (100 MHz, CDCl_3_): δ = 24.4, 25.7, 26.3, 26.4, 27.3, 28.1, 40.7, 53.5, 56.3, 63.6, 64.6, 71.2, 73.1, 74.2, 75.3, 76.5, 77.9, 78.0, 79.0, 103.7, 105.4, 108.3, 110.31, 110.34, 127.0, 127.3, 127.4, 127.5, 128.8, 129.7, 132.9, 139.6, 139.7, 140.2, 140.6, 171.3 ppm.

Compounds **3f–i** (0.25 mmol) were dissolved in [HBF_4_.(Et)_2_O]/H_2_O/dry CH_3_CN (2.1 mL, 1:5:500) and the mixture was stirred at 30 °C (0 °C in the case of **3g**) for 3 h, and filtered. Purification of the solids by trituration (petroleum ether) and lyophilisation gave **4f–i** as white solids.

6’-*O*-(2-Phenylethanoyl)-4-*O*-(β-d-galactopyranosyl)-d-glucopyranose (lactose phenyl acetate, **URB1419**) (**4f**)

Yield = 66%. MS (ESI): 478 [M + NH_4_]^+^, 483 [M + Na]^+^, 459 [M–H]^−^. ^1^H NMR (400 MHz, DMSO): δ = 3.21–3.24 (m, 1H, H^2^), 3.30–3.41 (m, 3H, H^4^, H^2’^, H^3’^), 3.59–3.68 (m, 4H, H^3^, H^6a^, H^6b^, H^4’^), 3.70 (s, 2H, CH_2_), 3.72–3.77 (m, 2H, H^5^, H^5’^), 4.11 (dd, 1H, *J*_H6’b-H5’_ = 4.0 Hz, *J*_H6’b-H6’a_ = 11.2 Hz, H^6’b^), 4.24 (dd, 1H, *J*_H6’a-H5’_ = 2.0 Hz, *J*_H6’a-H6’b_ = 11.2 Hz, H^6’a^), 4.25–4.27 (m, 1H, H^1’^), 4.34 (brs, 1H, OH), 4.47 (dd, 1H, *J*_OH6-H6a_ ≅ *J*_OH6-H6b_ = 6.0 Hz, OH^6^), 4.64 (brs, 1H, OH), 4.80 (brs, 1H, OH), 4.86 (brs, 1H, OH), 4.92 (dd, 1H, *J*_1-OH1_ ≅ *J*_H1-H2_ = 4.0 Hz, H^1^), 5.16 (brs, 1H, OH), 6.36 (d, 1H, *J*_OH1-H1_ = 4.0 Hz, OH^1^), 7.25–7.33 (m, 5H, ArH) ppm. ^13^C NMR (100 MHz, DMSO) δ = 31.2, 60.9, 64.3, 68.7, 70.2, 70.8, 71.7, 72.7, 72.8, 73.3, 81.7, 92.5, 104.1, 127.2, 128.8, 130.0, 134.7, 171.7 ppm.

6’-*O*-[2-(4-Phenyl)phenylethanoyl]-4-*O*-(β-d-galactopyranosyl)-d-glucopyranose (lactose biphenyl acetate, **URB1420**) (**4g**)

Yield = 72%. MS (*ESI*): 554 [M + NH_4_]^+^, 559 [M + Na]^+^, 535 [M–H]^−^. ^1^H NMR (400 MHz, DMSO): δ = 3.04 (dd, 1H, *J*_1_ ≅ *J*_2_ = 8.0 Hz), 3.23 (dd, 1H, *J*_1_ = 3.6 Hz, *J*_2_ = 9.6 Hz), 3.28–3.38 (m, 6H), 3.58–3.66 (m, 4H), 3.70–3.76 (m, 3H), 4.17–4.28 (m, 4H), 4.37 (d, 1H, *J* = 8.0 Hz), 4.94 (d, 1H, *J* = 3.6 Hz), 5.23 (s, 2H, CH_2_), 7.25–7.38 (m, 9H, ArH) ppm. ^13^C NMR (100 MHz, DMSO) δ = 56.2, 60.9, 64.7, 68.7, 70.2, 70.3, 70.7, 71.7, 72.7, 73.2, 75.2, 81.8, 92.6, 103.9, 127.47, 127.53, 128.9, 129.0, 129.1, 139.3, 139.4, 172.4 ppm.

6’-*O*-[2-(4-Phenyl)benzoyl]-4-*O*-(β-d-galactopyranosyl)-d-glucopyranose (lactose *p*-phenyl benzoate, **URB1421**) (**4h**)

Yield = 52%. MS (ESI): 540 [M + NH_4_]^+^, 545 [M + Na]^+^, 521 [M–H]^−^. ^1^H NMR (400 MHz, DMSO): δ = 3.22 (dd, 1H, *J*_1_ = 3.6 Hz, *J*_2_ = 9.6 Hz), 3.33–3.38 (m, 2H), 3.38–3.41 (m, 2H), 3.60–3.70 (m, 3H), 3.70–3.76 (m, 2H), 3.92 (dd, 1H, *J*_1_ = 4.0 Hz, *J*_2_ = 8.5 Hz), 4.26–4.36 (m, 3H), 4.48–4.56 (m, 2H), 4.91 (d, 1H, *J* = 4.0 Hz), 7.42–7.46 (m, 1H, ArH), 7.50–7.54 (m, 2H, ArH), 7.74–7.77 (m, 2H, ArH), 7.82–7.85 (m, 2H, ArH), 8.14–8.18 (m, 2H, ArH) ppm. ^13^C NMR (100 MHz, DMSO) δ = 60.8, 64.8, 68.9, 70.2, 70.8, 71.7, 72.8, 73.1, 73.3, 81.3, 92.6, 104.0, 127.3, 127.5, 128.8, 128.9, 129.6, 130.7, 139.4, 145.2 166.1 ppm.

6’-*O*-[2-(4,4’-Biphenyl)phenylethanoyl]-4-*O*-(β-d-galactopyranosyl)-d-glucopyranose (lactose triphenyl acetate, **URB1422**) (**4i**)

Yield = 58%. MS (ESI): 478 [M + NH_4_]^+^, 483 [M + Na]^+^, 459 [M–H]^−^. ^1^H NMR (400 MHz, DMSO): δ = 3.25 (dd, 1H, *J*_1_ = 3.2 Hz, *J*_2_ = 9.6 Hz), 3.30–3.42 (m, 4H), 3.51 (brs, 1H), 3.61–3.68 (m, 4H), 3.72–3.80 (m, 4H), 4.13 (dd, 1H, *J*_1_ = 3.2 Hz, *J*_2_ = 11.6 Hz), 4.27–4.39 (m, 4H), 4.93 (d, 1H, *J* = 3.2 Hz), 7.39–7.51 (m, 5H, ArH), 7.68–7.72 (m, 8H, ArH) ppm. ^13^C NMR (100 MHz, DMSO) δ = 60.9, 64.4, 68.7, 70.2, 70.8, 71.8, 72.7, 72.9, 73.3, 81.8, 92.6, 104.2, 126.96, 127.02, 127.6, 127.7, 128.0, 129.5, 130.7, 134.1, 138.5, 139.3, 139.5, 140.1, 171.6 ppm.

### 3.5. Synthesis of Triphenylacetic Acid *(**1i**)*

The synthetic procedure to obtain **1i** and all the intermediates is described in the Supplementary Materials (Scheme S1).

### 3.6. Bacterial Strains and Culture Conditions

*Escherichia coli* O157:H7 ATCC 35150, *Enterococcus faecalis* ATCC 29212, *Listeria monocytogenes* ATCC 7644, *Klebsiella pneumoniae* ATCC 13833, *Pseudomonas aeruginosa* ATCC 9027, Staphylococcus aureus ATCC 43387, *Salmonella enteritidis* ATCC 13076 and *Candida albicans* ATCC 14053 were the reference human pathogens used in this study (American Type Culture Collection, USA). All strains were maintained in Tryptic Soy Agar (TSA, Oxoid, Milan, Italy) at 37 °C, while *C. albicans* ATCC 14053 was grown in Sabouraud Dextrose Agar (SDA, Oxoid). All stock cultures were kept at −80 °C in nutrient broth with glycerol 15%.

### 3.7. Determination of MIC

The MICs of the tested compounds were determined by standard microdilution method according to the National Committee for Clinical Laboratory Standards (NCCLS), document M100-S12 method. First, each compound (5 mg) was dissolved in DMSO of biological grade (stock solutions) (1 mL). Several colonies of each bacterial strain were inoculated in sterile Mueller Hinton Broth (MHB, Oxoid) (10 mL) and incubated at 37 °C for 18–24 h. At the end, each bacterial suspension was adjusted to about 106 cfu/mL at nanomolar concentration (OD610 0.13–0.15) and 100 µL of that was added in wells of the 96-well plate, together with the appropriate volumes of the test solutions (1–256 µg/mL). Two rows were used for positive (bacteria alone) and negative controls (MHB alone), respectively. Gentamicin (0.125–128 µg/mL), fluconazole (0.125–128 µg/mL) (Sigma) and standard preservative mixture (methylparaben and propylparaben, ratio 9:1) (ACEF, Piacenza, Italy) (0.5–1024 µg/mL) were added as internal controls. Preliminary assays with DMSO were carried out to exclude its possible bacteriostatic and/or bactericidal activity; in any case, the volume of DMSO added in each well never exceeded 5% (*v*/*v*) of the final total volume. The experiments were performed in duplicate.

### 3.8. Formation of the Biofilm on 24-Well Polystyrene Plates

Selected food-borne pathogens, such as *E. coli* O157:H7 ATCC 35150, *L. monocytogenes* ATCC 7644, *S. aureus* ATCC 43387 and *S. enteritidis* ATCC 13076 were used. Each microorganism was inoculated in TSB (20 mL) and incubated overnight at 37 °C. After incubation, the optical density of each bacterial suspension was adjusted to about 106 cfu/mL (OD 610); then, 200 µL of each suspension were inoculated in 24-well polystyrene plates with the corresponding amount of each selected compound at relative MIC and 2× MIC values (final volume 1 mL/well). Two wells for each pathogen were inoculated with bacteria in TSB as controls. The plates were then incubated for 24 h, 48 h and 5 days at 37 °C to allow for biofilms development.

### 3.9. Evaluation of the Antibiofilm Activity of Sugar-Based Esters at Different Stage of Biofilm Formation

The antibiofilm activity of the selected compounds (glucose C10, mannose C10, lactose C10 and lactose biphenylacetate) was assessed at different times of development (24 h, 48 h and 96 h) of *E. coli* O157:H7 ATCC 35150, *L. monocytogenes* ATCC 7644, *S. aureus* ATCC 43387 and *S. enteritidis* ATCC 13076. At each time point, the inhibition of the biofilm formation was assessed by modified Crystal Violet (CV) staining [50]. As first, the planktonic cells were gently aspired, and then the formed biofilms were fixed with 99% methanol. Plates were washed twice with phosphate buffer saline (PBS) and air-dried, and then the biofilms were covered with CV 0.1% (*v*/*v*) for 15 min. The samples were washed again with PBS and air-dried. The remaining CV was dissolved in 85% ethanol (15 min at room temperature) and, finally, 200 µL of substance was transferred from each dish to a 96-well plate for the Multiscan Ex Microplate Reader (Thermo Scientific, Waltham, MA, USA) spectrophotometry at 570 nm. Each datapoint was averaged from at least eight replicate wells. The experiments were performed two times using independent cultures.

### 3.10. Caco-2 Cell Culture

Caco-2 cell line (i.e., human colon carcinoma) was obtained from the American Type Culture Collection (ATCC, Rockville, MD, USA). Caco-2 cells were grown routinely in 25 cm^2^ flasks containing DMEM (6 mL) supplemented with 10% FCS, 1% of non-essential amino acids and 1% of antibiotics (penicillin and streptomycin) at 37 °C with 5% CO_2_. At confluence, Caco-2 cells were treated with trypsin and seeded at a ratio of 2 × 104 cells/mL in new flasks. The plates were then incubated at 37 °C in 5% CO_2_ for five days to allow cell differentiation.

### 3.11. MTT Cell Proliferation Colorimetric Assay

The effect of the tested compounds on cell viability was measured using the MTT colorimetric assay (Millipore Corp., Burlington, MA, USA). Caco-2 cells were cultured on 96-well plates in DMEM for 48 h. Afterwards, the culture medium was replaced with DMEM containing glucose C10, mannose C10, lactose C10 and lactose biphenylacetate at different concentrations (0.01, 0.05, 0.1, 0.5, 1 and 2 mM). DMSO and PBS were used as a positive and negative control, assuming 100% and 0% cell death, respectively. Cells were incubated in 5% CO_2_ incubator at 37 °C for 24 h. Thereafter, the assay was performed according to the manufacturer’s instructions, with four replies for each sample. Percentage of viable cells was calculated using untreated cells as a control, with 100% cell viability. The percentage of viable cells was plotted against the concentrations of each tested compound in a dose–response model.

### 3.12. Statistical Analysis

Statistical analysis was performed using Prism version 5.0 (GraphPad Inc., San Diego, CA, USA). The assumptions for parametric test were cheeked prior to carry out the statistical analysis. *p* values < 0.05 were considered to be statistically significant.

## 4. Conclusions

Sugar-based monoesters made from glucose, mannose or lactose, and hydrophobic linear or aromatic acids were designed, synthesized, and evaluated as antimicrobial and antibiofilm agents. Specifically, glucose, mannose and lactose were coupled with saturated fatty acid chains C8, C10, C12, C14 and C16, while lactose was also esterified with aromatics tails.

The ability of these compounds to interact with a set of different microorganisms was assessed, obtaining MICs values ranging from 128 to >256 µg/mL. Successively, the antibiofilm activity of most relevant compounds (glucose C10, mannose C10, lactose C10 and lactose biphenylacetate) was evaluated at different times of development (24 h, 48 h and 5 days) of representative food-borne pathogens, such as *E. coli* O157:H7 ATCC 35150, *L. monocytogenes* ATCC 7644, *S. aureus* ATCC 43387 and *S. enteritidis* ATCC 13076. The antibiofilm activity trend was: Mannose C10 > lactose biphenylacetate > glucose C10 > lactose C10. In particular, mannose C10 and lactose biphenylacetate showed increased percentages of biofilm formation inhibition up to 96 h (97.2% and 92.0%, respectively, in the case of *E. coli* O157:H7 ATCC 35150). Interestingly, at MICs values, no toxicity for the selected surfactants was observed on the human colorectal cell line using the MTT assay. For all the obtained results, the sugar-based monoesters here presented, in particular mannose C10 and lactose biphenylacetate, could be proposed as possible biocompatible and safe preservatives for pharmaceutical, food and other industrial applications.

## Figures and Tables

**Table 1:** Calculated physico-chemical properties of the sugar-based ester surfactants.

Entry	Sugar Ester	MW	cHLB ^a^	cLogP ^b^	cTPSA ^c^
1	Glucose C8	306.4	8.9	0.59	116.5
2	Glucose C10	334.4	8.1	1.43	116.5
3	Glucose C12	362.5	7.5	2.26	116.5
4	Glucose C14	390.5	7.0	3.20	116.5
5	Glucose C16	418.6	6.5	3.93	116.5
6	Mannose C8	306.4	8.9	0.59	116.5
7	Mannose C10	334.4	8.1	1.43	116.5
8	Mannose C12	362.5	7.5	2.26	116.5
9	Mannose C14	390.5	7.0	3.20	116.5
10	Mannose C16	418.6	6.5	3.93	116.5
11	Lactose C8	468.5	12.7	−1.15	195.6
12	Lactose C10	496.6	12.0	−0.31	195.6
13	Lactose C12	524.6	11.4	0.52	195.6
14	Lactose C14	552.7	10.8	1.36	195.6
15	Lactose C16	580.1	10.3	2.19	195.6
16	Lactose phenylacetate	460.4	12.9	−2.05	195.6
17	Lactose *p*-phenylbenzoate	522.5	11.4	−0.32	195.6
18	Lactose biphenylacetate	536.5	11.1	−0.37	195.6
19	Lactose triphenylacetate	612.6	9.7	1.30	195.6

^a^ Calculated HLB (cHLB) by Griffin’s method for non-ionic surfactants [25] [cHLB = 20× (MW hydrophilic portion/MW)]. cHLB values for glucose esters (entry 1–5) perfectly matched with the measured values [22]. ^b^ Calculated LogP (cLogP) (by OSIRIS Property Explorer) [26]. ^c^ Calculated TPSA (cTPSA) (by OSIRIS Property Explorer) [26].

**Table 2:** MIC values (µg/mL) of the tested sugar fatty acids against selected microorganisms. ^a^ Fluconazole was used as control for *C. albicans* ATCC 10231.

	*E. coli* O157:H7 ATCC 35150	*E. faecalis* ATCC 29212	*L. monocytogenes* ATCC 7644	*K. pneumoniae* ATCC 13883	*P. aeruginosa* ATCC 9027	*S. aureus* ATCC 43387	*S. enteritidis* ATCC 13076	*C. albicans* ATCC 10231
Glucose C8	256	256	256	>256	>256	>256	256	256
Glucose C10	256	256	256	256	256	256	256	256
Glucose C12	>256	>256	>256	>256	>256	>256	>256	>256
Glucose C14	>256	>256	>256	>256	>256	>256	>256	>256
Glucose C16	>256	>256	>256	>256	>256	>256	>256	>256
Mannose C8	>256	>256	>256	>256	>256	>256	>256	>256
Mannose C10	256	256	256	256	256	256	256	256
Mannose C12	256	256	256	256	256	256	256	256
Mannose C14	>256	256	>256	256	256	>256	>256	256
Mannose C16	>256	256	>256	256	>256	>256	>256	>256
Lactose C8	256	256	256	256	>256	256	256	256
Lactose C10	256	128	256	256	256	256	256	128
Lactose C12	>256	>256	>256	>256	>256	>256	>256	256
Lactose C14	>256	256	>256	>256	>256	>256	>256	256
Lactose C16	>256	>256	>256	>256	>256	>256	>256	256
Lactose phenylacetate	256	256	256	256	>256	>256	256	256
Lactose biphenylacetate	256	256	256	256	256	256	256	256
Lactose *p*-phenylbenzoate	256	256	256	256	256	256	256	256
Lactose triphenylacetate	256	256	256	256	256	256	256	256
Gentamicin	16	64	8	8	16	16	4	1 ^a^
Parabens	>1024	>1024	>1024	>1024	>1024	>1024	>1024	>1024

**Table 3:** Percentage values of biofilm formation inhibition after 24 h, 48 h and 96 h of incubation with selected sugar fatty acids at their relative MIC and 2× MIC values. In grey are indicated the percentage values > 30%, herein considered as an index of antibiofilm activity.

	24 h		48 h		5 Days	
	MIC	2× MIC	MIC	2× MIC	MIC	2× MIC
	Glucose C10		Glucose C10		Glucose C10	
*E. coli* O157:H7 ATCC 35150	10.77%	18.71%	18.27%	25.94%	35.46%	37.90%
*L. monocytogenes* ATCC 7644	9.61%	14.17%	12.69%	21.95%	18.04%	34.58%
*S. aureus* ATCC 43387	8.42%	16.98%	13.18%	23.05%	18.01%	23.57%
*S. enteritidis* ATCC 13076	14.05%	41.27%	9.97%	20.66%	36.94%	46.28%
	Mannose C10		Mannose C10		Mannose C10	
*E. coli* O157:H7 ATCC 35150	38.30%	66.74%	58.19%	84.38%	81.13%	97.24%
*L. monocytogenes* ATCC 7644	46.19%	62.32%	47.15%	65.45%	49.05%	91.80%
*S. aureus* ATCC 43387	41.49%	99.58%	48.72%	99.88%	42.16%	54.61%
*S. enteritidis* ATCC 13076	23.78%	98.54%	32.16%	42.71%	37.04%	38.61%
	Lactose C10		Lactose C10		Lactose C10	
*E. coli* O157:H7 ATCC 35150	7.54%	11.69%	9.66%	26.75%	35.46%	43.60%
*L. monocytogenes* ATCC 7644	9.61%	16.64%	13.53%	27.76%	15.38%	48.86%
*S. aureus* ATCC 43387	6.72%	18.13%	21.13%	24.66%	41.40%	55.26%
*S. enteritidis* ATCC 13076	3.55%	12.79%	3.61%	14.81%	11.00%	21.48%
	Lactose biphenylacetate		Lactose biphenylacetate		Lactose biphenylacetate	
*E. coli* O157:H7 ATCC 35150	40.10%	46.99%	61.31%	65.65%	85.21%	91.97%
*L. monocytogenes* ATCC 7644	19.42%	27.12%	26.98%	34.66%	76.14%	78.77%
*S. aureus* ATCC 43387	15.35%	39.00%	22.46%	40.27%	33.03%	53.91%
*S. enteritidis* ATCC 13076	20.58%	38.35%	45.41%	52.22%	48.12%	53.27%

## Supplementary Materials

The following are available online at https://www.mdpi.com/1424-8247/12/4/186/s1, Supplementary material: 1. Characterization of glucose and mannose fatty acid ester derivatives **7b–e** and **8b–e**; 2. Scheme S1: Synthesis of triphenylacetic acid (**1i**).
