# Supplementary material for: Synthesis and Evaluation of Saccharide-Based Aliphatic and Aromatic Esters as Antimicrobial and Antibiofilm Agents
Source: Pharmaceuticals (Basel). 2019 Dec 17;12(4):186. doi: 10.3390/ph12040186 (PMC6958352; doi:10.3390/ph12040186)
Supplement: Supplementary file 1 [file pharmaceuticals-12-00186-s001.pdf]

# Supplementary material

## Synthesis and evaluation of saccharide-based aliphatic and aromatic esters as antimicrobial and antibiofilm agents

Raffaella Campana[a], Alessio Merli[a], Michele Verboni[a], Francesca Biondo[a], Gianfranco Favi[a], Andrea Duranti[a*] and Simone Lucarini[a*]

[a]Department of Biomolecular Sciences, University of Urbino Carlo Bo, 61029, Urbino (PU), Italy.

* Corresponding authors.

*E-mail addresses*: andrea.duranti@uniurb.it (A. Duranti) and simone.lucarini@uniurb.it (S. Lucarini)

**Table of Contents**


| | | |
|---|---|---|
| 1. | Characterization of glucose and mannose fatty acid ester derivatives **7b–e** and **8b–e** | S2 |
| 2. | Scheme S1: Synthesis of triphenylacetic acid (**1i**). | S6 |
| 3. | References | S7 |

## 1. Characterization of glucose and mannose fatty acid ester derivatives 7b–e and 8a–e.

**6-*O*-Decanoyl-D-mannopyranose (mannose caprate, URB1390) (7b) [1]**

Yield = 59%, α/β = 1:0.5. $^1$H NMR (400 MHz, DMSO): δ = 0.86 (t, 3H+1.5H, $J$ = 6.5 Hz, $CH_3$), 1.24–1.26 (m, 12H+6H), 1.49–1.53 (m, 2H+1H, $OCCH_2$*$CH_2$*), 2.25–2.31 (m, 2H+1H, OC*$CH_2$*$CH_2$), 3.21–3.32 (m, 1.5H, $H^{3\beta}$, $H^{4\beta}$, $H^{5\beta}$), 3.35–3.41 (m, 1H, $H^{4\alpha}$), 3.49–3.56 (m, 2H+0.5H, $H^{2\alpha}$, $H^{3\alpha}$, $H^{2\beta}$), 3.70 (ddd, 1H, $J_{H5\alpha\text{-}H6b\alpha}$ = 1.5 Hz, $J_{H5\alpha\text{-}H6a\alpha}$ = 7.0 Hz, $J_{H5\alpha\text{-}H4\alpha}$ = 9.0 Hz, $H^{5\alpha}$), 3.94–4.01 (m, 1H+0.5H, $H^{6a\alpha}$, $H^{6\alpha\beta}$), 4.27–4.32 (m, 1H+0.5H, $H^{6b\alpha}$, $H^{6b\beta}$), 4.53–4.59 (m, 1H+1H, $OH^{2\alpha}$, $H^{1\beta}$, $OH^{2\beta}$), 4.63 (d, 1H, $J_{OH3\alpha\text{-}H3\alpha}$ = 4.5 Hz, $OH^{3\alpha}$), 4.66 (d, 0.5H, $J_{OH3\beta\text{-}H3\beta}$ = 5.5 Hz, $OH^{3\beta}$), 4.86 (dd, 1H, $J_{H1\alpha\text{-}H2\alpha} \approx J_{H1\alpha\text{-}OH1\alpha}$ = 4.5 Hz, $H^{1\alpha}$), 4.89 (d, 1H, $J_{OH4\alpha\text{-}H4\alpha}$ = 6.0 Hz, $OH^{4\alpha}$), 4.94 (d, 0.5H, $J_{OH4\beta\text{-}H4\beta}$ = 5.0 Hz, $OH^{4\beta}$), 6.26 (d, 0.5H, $J_{OH1\beta\text{-}H1\beta}$ = 8.0 Hz, $OH^{1\beta}$), 6.37 (d, 1H, $J_{OH1\alpha\text{-}H1\alpha}$ = 5.0 Hz, $OH^{1\alpha}$) ppm. $^{13}$C NMR (100 MHz, DMSO): δ = 14.4 (1.5C), 22.6 (1.5C), 24.9 (1.5C), 28.9 (1.5C), 29.1 (1.5C), 29.2 (1.5C), 29.3 (1.5C), 31.7 (1.5C), 33.9 (1.5C), 64.7 (C6, 1.5C), 67.2 (C5, 0.5C), 67.6 (C5, 1C), 70.8 (C4, 1C), 70.9 (C3, 1C), 71.8 (C2, 1C), 72.0 (C2, 0.5C), 73.9 (C4, 0.5), 74.5 (C3, 0.5C), 94.5 (C1, 1C), 94.6 (C1, 0.5C), 173.5 (CO, 1.5C) ppm.

**6-*O*-Dodecanoyl-D-mannopyranose (mannose laurate, URB1380) (7c)** [1]

Yield = 12%, α/β = 1:0.5. $^1$H NMR (400 MHz, DMSO): δ = 0.86 (t, 3H+1.5H, $J$ = 7.0 Hz, $CH_3$), 1.24–1.26 (m, 16H+8H), 1.49–1.53 (m, 2H+1H, $OCCH_2$*$CH_2$*), 2.25–2.30 (m, 2H+1H, OC*$CH_2$*$CH_2$), 3.21–3.33 (m, 1.5H, $H^{3\beta}$, $H^{4\beta}$, $H^{5\beta}$), 3.35–3.41 (m, 1H, $H^{4\alpha}$), 3.49–3.55 (m, 2H+0.5H, $H^{2\alpha}$, $H^{3\alpha}$, $H^{2\beta}$), 3.70 (ddd, 1H, $J_{H5\alpha\text{-}H6b\alpha}$ = 1.5 Hz, $J_{H5\alpha\text{-}H6a\alpha}$ = 7.0 Hz, $J_{H5\alpha\text{-}H4\alpha}$ = 9.0 Hz, $H^{5\alpha}$), 3.94–4.02 (m, 1H+0.5H, $H^{6a\alpha}$, $H^{6a\beta}$), 4.27–4.32 (m, 1H+0.5H, $H^{6b\alpha}$, $H^{6b\beta}$), 4.53–4.59 (m, 1H+1H, $OH^{2\alpha}$, $H^{1\beta}$, $OH^{2\beta}$), 4.63 (d, 1H, $J_{OH3\alpha\text{-}H3\alpha}$ = 4.0 Hz, $OH^{3\alpha}$), 4.66 (d, 0.5H, $J_{OH3\beta\text{-}H3\beta}$ = 5.5 Hz, $OH^{3\beta}$), 4.86 (dd, 1H, $J_{H1\alpha\text{-}H2\alpha} \approx J_{H1\alpha\text{-}OH1\alpha}$ = 4.5 Hz, $H^{1\alpha}$), 4.89 (d, 1H, $J_{OH4\alpha\text{-}H4\alpha}$ = 5.5 Hz, $OH^{4\alpha}$), 4.94 (d, 0.5H, $J_{OH4\beta\text{-}H4\beta}$ = 5.0 Hz, $OH^{4\beta}$), 6.26 (d, 0.5H, $J_{OH1\beta\text{-}H1\beta}$ = 8.5 Hz, $OH^{1\beta}$), 6.38 (d, 1H, $J_{OH1\alpha\text{-}H1\alpha}$ = 4.5 Hz, $OH^{1\alpha}$) ppm. $^{13}$C NMR (100 MHz, DMSO): δ = 14.4 (1.5C), 22.6 (1.5C), 24.9 (1.5C), 28.9 (1.5C), 29.17 (1.5C), 29.20 (1.5), 29.4

(1.5), 29.5, 31.8 (1.5C), 33.88 (0.5C), 33.93 (1C), 64.6 (C6, 0.5C), 64.7 (C6, 1C), 67.3 (C5, 0.5C), 67.6 (C5, 1C), 70.8 (C4, 1C), 70.9 (C3, 1C), 71.8 (C2, 1C), 72.0 (C2, 0.5C), 73.9 (C4, 0.5), 74.5 (C3, 0.5C), 94.5 (C1, C), 94.6 (C1, 0.5C), 173.5 (CO, 1.5C) ppm.

**(2*R*,3*S*,4*S*,5*S*,6*S*)-6-*O*-Tetradecanoyl-D-mannopyranose** and **(2*R*,3*S*,4*S*,5*S*,6*R*)-6-*O*-tetradecanoyl-D-mannopyranose (mannose myristate, URB1381) (7d)** [1]

Yield = 60%, α/β = 1:0.5. $^{1}$H NMR (400 MHz, DMSO): δ = 0.86 (t, 3H+1.5H, $J$ = 6.5 Hz, $CH_3$), 1.24–1.26 (m, 20H+10H), 1.50–1.53 (m, 2H+1H, $OCCH_2$*$CH_2$*), 2.26–2.31 (m, 2H+1H, OC*$CH_2$*$CH_2$), 3.21–3.33 (m, 1.5H, $H^{3\beta}$, $H^{4\beta}$, $H^{5b}$), 3.35–3.41 (m, 1H, $H^{4\alpha}$), 3.49–3.55 (m, 2H+0.5H, $H^{2\alpha}$, $H^{3\alpha}$, $H^{2\beta}$), 3.70 (ddd, 1H, $J_{H5\alpha\text{-}H6b\alpha}$ = 1.5 Hz, $J_{H5\alpha\text{-}H6a\alpha}$ = 7.0 Hz, $J_{H5\alpha\text{-}H4\alpha}$ = 9.0 Hz, $H^{5\alpha}$), 3.94–4.02 (m, 1H+0.5H, $H^{6a\alpha}$, $H^{6a\beta}$), 4.27–4.32 (m, 1H+0.5H, $H^{6b\alpha}$, $H^{6b\beta}$), 4.53–4.59 (m, 1H+1H, $OH^{2\alpha}$, $H^{1\beta}$, $OH^{2\beta}$), 4.63 (d, 1H, $J_{OH3\alpha\text{-}H3\alpha}$ = 4.0 Hz, $OH^{3\alpha}$), 4.66 (d, 0.5H, $J_{OH3\beta\text{-}H3\beta}$ = 5.5 Hz, $OH^{3\beta}$), 4.86 (dd, 1H, $J_{H1\alpha\text{-}H2\alpha}$ ≅ $J_{H1\alpha\text{-}OH1\alpha}$ = 4.5 Hz, $H^{1\alpha}$), 4.89 (d, 1H, $J_{OH4\alpha\text{-}H4\alpha}$ = 5.5 Hz, $OH^{4\alpha}$), 4.94 (d, 0.5H, $J_{OH4\beta\text{-}H4\beta}$ = 5.0 Hz, $OH^{4\beta}$), 6.26 (d, 0.5H, $J_{OH1\beta\text{-}H1\beta}$ = 8.5 Hz, $OH^{1\beta}$), 6.38 (d, 1H, $J_{OH1\alpha\text{-}H1\alpha}$ = 4.5 Hz, $OH^{1\alpha}$) ppm. $^{13}$C NMR (100 MHz, DMSO): δ = 14.4 (1.5C), 22.6 (1.5C), 24.9 (1.5C), 29.0 (1.5C), 29.18 (1.5C), 29.21 (1.5), 29.38 (1.5), 29.5, 31.8 (1.5C), 33.9 (1.5C), 64.7 (C6, 1.5C), 67.3 (C5, 0.5C), 67.6 (C5, 1C), 70.8 (C4, 1.5C), 71.8 (C2, 1C), 72.0 (C2, 0.5C), 74.0 (C4, 0.5), 74.5 (C3, 0.5C), 94.5 (C1, C), 94.6 (C1, 0.5C), 173.4 (CO, 1.5C) ppm.

**(2*R*,3*S*,4*S*,5*S*,6*S*)-6-*O*-Esadecanoyl-D-mannopyranose** and **(2*R*,3*S*,4*S*,5*S*,6*R*)-6-*O*-esadecanoyl-D-mannopyranose (mannose palmitate, URB1382) (7e)** [2]

Yield = 32%, α/β = 1:0.8. MS (ESI): 417.5 [M-H], 436.4 [M+$NH_4$], 441.4 [M+Na]. $^{1}$H NMR (400 MHz, DMSO): δ = 0.86 (t, 3H+2.4H, $J$ = 6.5 Hz, $CH_3$), 1.24–1.26 (m, 24H+10H), 1.50–1.53 (m, 2H+1.6H, $OCCH_2$*$CH_2$*), 2.25–2.30 (m, 2H+1.6H, OC*$CH_2$*$CH_2$), 3.24–3.32 (m, 0.8x3H, $H^{3\beta}$, $H^{4\beta}$, $H^{5\beta}$), 3.34–3.39 (m, 1H, $H^{4\alpha}$), 3.51–3.56 (m, 2H+0.8H, $H^{2\alpha}$, $H^{3\alpha}$, $H^{2\beta}$), 3.71 (ddd, 1H, $J_{H5\,\alpha\text{-}H6b\,\alpha}$ = 1.5 Hz, $J_{H5\,\alpha\text{-}H6a\,\alpha}$ = 7.0 Hz, $J_{H5\,\alpha\text{-}H4\,\alpha}$ = 9.0 Hz, $H^{5\alpha}$), 3.95–4.02 (m, 1H+0.8H, $H^{6a\alpha}$, $H^{6a\beta}$), 4.28–4.33 (m, 1H+0.8H, $H^{6b\alpha}$, $H^{6b\beta}$), 4.51–4.53 (m, 1H+0.8H, $OH^{2\alpha}$, $H^{1\beta}$), 4.57–4.59 (m, 1H+0.8H, $OH^{3\alpha}$, $OH^{2\beta}$),

4.62 (d, 0.8H, $J_{OH3\beta-H3\beta}$ = 4.5 Hz, $OH^{3\beta}$), 4.84–4.87 (m, 2H, $H^{1\alpha}$, $OH^{4\alpha}$), 4.90 (d, 0.8H, $J_{OH4\beta-H4\beta}$ = 4.5 Hz, $OH^{4\beta}$), 6.22 (d, 0.8H, $J_{OH1\beta-H1\beta}$ = 8.5 Hz, $OH^{1\beta}$), 6.35 (d, 1H, $J_{OH1\alpha-H1\alpha}$ = 4.5 Hz, $OH^{1\alpha}$) ppm. $^{13}C$ NMR (100 MHz, DMSO): δ = 14.4 (1.8C), 22.6 (1.8C), 24.9 (1.8C), 29.0 (1.8C), 29.16 (1.8C), 29.20 (1.8C), 29.4 (1.8C), 29,47, 29.51, 31.8 (1.8C), 33.88 (0.8C), 33.93 (1C), 64.7 (C6, 1.8 C), 67.3 (C5, 0.8C), 67.6 (C5, 1C), 70.8 (C4, 1.8C), 71.8 (C3, 1C), 72.0 (C2, 1C), 74.0 (C4, 0.8), 74.5 (C3, 0.8C), 94.5 (C1, 1C), 94.6 (C1, 0.8C), 173.4 (CO, 1.8C) ppm.

**6-*O*-Decanoyl-D-glucopyranose (glucose caprate**, **URB1385**) **(8b)** [3]

Yield = 15%. $^{1}H$ NMR (400 MHz, DMSO): δ = 0.86 (t, 3H, $J$ = 7.0 Hz, $CH_3$), 1.22–1.28 (m, 12H), 1.48–1.54 (m, 2H, $OCCH_2\mathit{CH_2}$), 2.28 (t, 2H, $J$ = 7.5 Hz, $OC\mathit{CH_2}CH_2$), 3.03 (ddd, 1H, $J_{H4-OH4}$ = 6.0 Hz, $J_{H4-H3}$ = 9.0 Hz, $J_{H4-H5}$ = 9.5 Hz, $H^4$), 3.13 (ddd, 1H, $J_{H2-H1}$ = 4.0 Hz, $J_{H2-OH2}$ = 6.5 Hz, $J_{H2-H3}$ = 9.0 Hz, $H^2$), 3.43 (ddd, 1H, $J_{H3-OH3}$ = 5.0 Hz, $J_{H3-H2}$ ≅ $J_{H3-H4}$ = 9.0 Hz, $H^3$), 3.77 (ddd, 1H, $J_{H5-H6b}$ = 2.0 Hz, $J_{H5-H6a}$ = 6.0 Hz, $J_{H5-H4}$ = 9.5 Hz, $H^5$), 3.99 (dd, 1H, $J_{H6a-H5}$ = 6.0 Hz, $J_{H6a-H6b}$ = 12.0 Hz, $H^{6a}$), 4.27 (dd, 1H, $J_{H6b-H5}$ = 2.0 Hz, $J_{H6b-H6a}$ = 12.0 Hz, $H^{6b}$), 4.54 (d, 1H, $J_{OH2-H2}$ = 6.5 Hz, $OH^2$), 4.76 (d, H, $J_{OH3-H3}$ = 5.0 Hz, $OH^3$), 4.90 (dd, 1H, $J_{H1-H2}$ = 4.0 Hz, $J_{H1-OH1}$ = 4.5 Hz, $H^1$), 5.05 (d, 1H, $J_{OH4-H4}$ = 6.0 Hz, $OH^4$), 6.35 (d, 1H, $J_{OH1-H1}$ = 4.5 Hz, $OH^1$) ppm. $^{13}C$ NMR (100 MHz, DMSO): δ = 14.4, 22.6, 24.9, 28.9, 29.1, 29.2, 29.3, 31.7, 33.9, 64.3 (C6), 69.6 (C5), 71.0 (C4), 72.7 (C2), 73.3 (C3), 92.7 (C1), 173.4 (CO) ppm.

**6-*O*-Dodecanoyl-D-glucopyranose (glucose laurate**, **URB1384**) **(8c)** [3]

Yield = 15%. $^{1}H$ NMR (400 MHz, DMSO): δ = 0.86 (t, 3H, $J$ = 7.0 Hz, $CH_3$), 1.23–1.26 (m, 16H), 1.49–1.52 (m, 2H, $OCCH_2\mathit{CH_2}$), 2.27 (t, 2H, $J$ = 6.5 Hz, $OC\mathit{CH_2}CH_2$), 3.04 (ddd, 1H, $J_{H4-OH4}$ = 5.5 Hz, $J_{H4-H3}$ = 9.0 Hz, $J_{H4-H5}$ = 9.5 Hz, $H^4$), 3.12 (ddd, 1H, $J_{H2-H1}$ = 4.0 Hz, $J_{H2-OH2}$ = 6.5 Hz, $J_{H2-H3}$ = 9.0 Hz, $H^2$), 3.43 (ddd, 1H, $J_{H3-OH3}$ = 5.0 Hz, $J_{H3-H2}$ ≅ $J_{H3-H4}$ = 9.0 Hz, $H^3$), 3.76 (ddd, 1H, $J_{H5-H6b}$ = 1.5 Hz, $J_{H5-H6a}$ = 6.5 Hz, $J_{H5-H4}$ = 9.5 Hz, $H^5$), 3.99 (dd, 1H, $J_{H6a-H5}$ = 6.5 Hz, $J_{H6a-H6b}$ = 11.5 Hz, $H^{6a}$), 4.26 (dd, 1H, $J_{H6b-H5}$ = 1.5 Hz, $J_{H6b-H6a}$ = 11.5 Hz, $H^{6b}$), 4.51 (d, 1H, $J_{OH2-H2}$ = 6.5 Hz, $OH^2$), 4.73 (d, H, $J_{OH3-H3}$ = 5.0 Hz, $OH^3$), 4.89 (dd, 1H, $J_{H1-H2}$ = 4.0 Hz, $J_{H1-OH1}$ = 4.5 Hz, $H^1$), 5.02 (d, 1H, $J_{OH4-H4}$

= 5.5 Hz, $OH^4$), 6.32 (d, 1H, $J_{OH1-H1}$ = 4.5 Hz, $OH^1$) ppm. $^{13}$C NMR (100 MHz, DMSO): δ = 14.4, 22.6, 24.9, 28.9, 29.16, 29.18, 29.4, 29.5 (2C), 31.8, 33.9, 64.3 (C6), 69.6 (C5), 71.0 (C4), 72.7 (C2), 73.3 (C3), 92.8 (C1), 173.4 (CO) ppm.

**Tetradecanoyl-D-glucopyranose (glucose myristate, URB1386) (8d)** [3]

Yield = 10%. $^1$H NMR (400 MHz, DMSO): δ = 0.85 (t, 3H, $J$ = 7.0 Hz, $CH_3$), 1.21–1.28 (m, 20H), 1.47–1.54 (m, 2H, $OCCH_2\mathit{CH_2}$), 2.27 (t, 2H, $J$ = 6.5 Hz, $OC\mathit{CH_2}CH_2$), 3.03 (ddd, 1H, $J_{H4-OH4}$ = 5.5 Hz, $J_{H4-H3}$ = 9.0 Hz, $J_{H4-H5}$ = 9.5 Hz, $H^4$), 3.13 (ddd, 1H, $J_{H2-H1}$ = 4.0 Hz, $J_{H2-OH2}$ = 6.5 Hz, $J_{H2-H3}$ = 9.0 Hz, $H^2$), 3.43 (ddd, 1H, $J_{H3-OH3}$ = 5.0 Hz, $J_{H3-H2} \cong J_{H3-H4}$ = 9.0 Hz, $H^3$), 3.77 (ddd, 1H, $J_{H5-H6b}$ = 1.5 Hz, $J_{H5-H6a}$ = 6.5 Hz, $J_{H5-H4}$ = 9.5 Hz, $H^5$), 3.99 (dd, 1H, $J_{H6a-H5}$ = 6.5 Hz, $J_{H6a-H6b}$ = 11.5 Hz, $H^{6a}$), 4.27 (dd, 1H, $J_{H6b-H5}$ = 1.5 Hz, $J_{H6b-H6a}$ = 11.5 Hz, $H^{6b}$), 4.55 (d, 1H, $J_{OH2-H2}$ = 6.5 Hz, $OH^2$), 4.79 (d, 1H, $J_{OH3-H3}$ = 5.0 Hz, $OH^3$), 4.89 (dd, 1H, $J_{H1-H2}$ = 4.0 Hz, $J_{H1-OH1}$ = 4.5 Hz, $H^1$), 5.06 (d, 1H, $J_{OH4-H4}$ = 5.5 Hz, $OH^4$), 6.36 (d, 1H, $J_{OH1-H1}$ = 4.5 Hz, $OH^1$) ppm.$^{13}$C NMR (100 MHz, DMSO): δ = 14.4, 22.6, 24.9, 28.9, 29.18, 29.19, 29.37, 29.48, 29.50, 29.52, 31.8, 33.9, 64.3 (C6), 69.6 (C5), 71.0 (C4), 72.7 (C2), 73.3 (C3), 92.8 (C1), 173.4 (CO) ppm.

**Esadecanoyl-D-glucopyranose (glucose palmitate, URB1387) (8e)** [3]

Yield = 5%. $^1$H NMR (400 MHz, DMSO): δ = 0.86 (t, 3H, $J$ = 7.0 Hz, $CH_3$), 1.19–1.28 (m, 24H), 1.47–1.53 (m, 2H, $OCCH_2\mathit{CH_2}$), 2.27 (t, 2H, $J$ = 7.5 Hz, $OC\mathit{CH_2}CH_2$), 3.04 (ddd, 1H, $J_{H4-OH4}$ = 5.5 Hz, $J_{H4-H3}$ = 9.0 Hz, $J_{H4-H5}$ = 9.5 Hz, $H^4$), 3.12 (ddd, 1H, $J_{H2-H1}$ = 4.0 Hz, $J_{H2-OH2}$ = 6.5 Hz, $J_{H2-H3}$ = 9.5 Hz, $H^2$), 3.43 (ddd, 1H, $J_{H3-OH3}$ = 4.5 Hz, $J_{H3-H2} \cong J_{H3-H4}$ = 9.5 Hz, $H^3$), 3.77 (ddd, 1H, $J_{H5-H6b}$ = 1.5 Hz, $J_{H5-H6a}$ = 6.0 Hz, $J_{H5-H4}$ = 9.0 Hz, $H^5$), 3.99 (dd, 1H, $J_{H6a-H5}$ = 6.0 Hz, $J_{H6a-H6b}$ = 11.5 Hz, $H^{6a}$), 4.27 (dd, 1H, $J_{H6b-H5}$ = 1.5 Hz, $J_{H6b-H6a}$ = 11.5 Hz, $H^{6b}$), 4.51 (d, 1H, $J_{OH2-H2}$ = 6.5 Hz, $OH^2$), 4.74 (d, H, $J_{OH3-H3}$ = 4.5 Hz, $OH^3$), 4.90 (dd, 1H, $J_{H1-H2} \cong J_{H1-OH1}$ = 4.0 Hz, $H^1$), 5.02 (d, 1H, $J_{OH4-H4}$ = 5.5 Hz, $OH^4$), 6.34 (d, 1H, $J_{OH1-H1}$ = 4.0 Hz, $OH^1$) ppm. $^{13}$C NMR (100 MHz, DMSO): δ = 14.4, 22.6, 24.9, 28.9, 29.16 (2C), 29.19, 29.36, 29.46 (2C), 29.5 (2C), 31.2, 31.8, 33.9, 64.3 (C6), 69.6 (C5), 71.0 (C4), 72.7 (C2), 73.3 (C3), 92.8 (C1), 173.4 (CO) ppm.

## 2. Synthesis of triphenylacetic acid (1i) (Scheme S1).

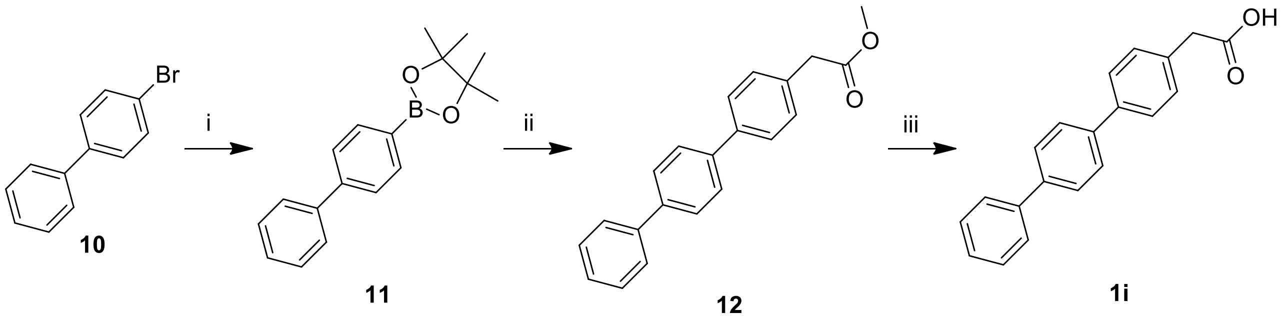


**Scheme 1S.** Reagents and conditions: (i) $B_2pin_2$, $Pd(dppf)Cl_2/CH_2Cl_2$, dppf, KOAc, dry dioxane, 80 °C, 16 h; ii) methyl 2-(4-bromophenyl)acetate, Pd(II)(dba)3, $PCy_3$, $K_3PO_4/H_2O$, dioxane:$H_2O$ 2:1, 75 °C, 16 h; iii) LiOH, MeOH:$H_2O$ 3:1, 60 °C, 5 h.

**Methyl 2-(4-bromophenyl)acetate (11)** [4]

A mixture of *p*-phenylbromobenzene (0.534 g, 2.29 mmol) (**10**), $B_2pin_2$ (1.635 g, 6.44 mmol), $Pd(dppf)Cl_2$ in $CH_2Cl_2$ (0.170 g, 0.21 mmol), dppf (0.083 g, 0.15 mmol) and KOAc (1.320 g, 13.43 mmol) in dry dioxane (11.6 mL) was stirred at 80 °C for 20 h, then filtered on Celite®, and extracted with EtOAc. The combined organic layers were washed with $H_2O$, dried ($Na_2SO_4$), and concentrated. The purification of the residue by column cromatography (cyclohexane/EtOAc 98:2) gave **11** as a white solid. Yield = 96%. $^1H$ NMR (400 MHz, $CDCl_3$): δ = 1.29 (s, 12H, 6 $CH_3$), 7.26–7.31 (m, 1H, ArH), 7.35–7.39 (m, 2H, ArH), 7.53–7.56 (m, 4H, ArH), 7.81–7.83 (m, 2H, ArH).

**Methyl 2-[(1,1':4',1"-triphenyl)-4-yl]acetate (12)** [4]

A $K_3PO_4$ 1,27 M aqueous solution (4 mL) was added to solution of **11** (0.999 g, 3.57 mmol), methyl 2-(4-bromophenyl)acetate (0.682 g, 2.98 mmol), $Pd_2(dba)_3$ (0.164 g, 0.18 mmol), $PCy_3$ (0.114 g, 0.41 mmol) in a mixture 2:1 dioxane/$H_2O$ (8 mL). The solution was stirred at 80 °C for 20 h, then filtered on Celite®, and extracted with EtOAc. The combined organic layers were washed with $H_2O$, dried ($Na_2SO_4$), and concentrated. The purification by column cromatography (cyclohexane/EtOAc 98:2 then petroleum ether/$Et_2O$ 95:5) gave **12** as a white solid. Yield = 47%. $^1H$ NMR (400 MHz, $CDCl_3$): δ = 3.62 (s, 2H, $CH_2$), 3.65 (s, 3H, $CH_3$), 7.29–7.31 (m, 3H, ArH), 7.34–7.41 (m, 2H, ArH), 7.53–

7.58 (m, 4H, ArH), 7.59–7.61 (m, 4H, ArH) ppm. $^{13}$C NMR (100 MHz, $CDCl_3$) δ = 40.8, 52.1, 127.0, 127.2, 127.3, 127.4, 127.5, 128.8, 129.7, 133.1, 139.6, 139.7, 140.2, 140.7, 172.0 ppm.

**2-[(1,1',4',1"-triphenyl)4-yl]acetic acid (1i)** [5]

A solution of **12** (0.420 g, 1.39 mmol) and LiOH (0.292 g, 6.95 mmol) in a mixture 3:1 MeOH/$H_2O$ (9.3 mL) was stirred at 60 °C for 5 h, then acidified with HCl 2N to pH = 2, and extracted with EtOAc. The combined organic layers were washed with $H_2O$, dried ($Na_2SO_4$), and concentrated. The purification by recrystallization from EtOAc gave **1i** as a white solid. Yield = 79%. Mp = 266-268 °C. MS (ESI): 289 $[M + H]^+$, 287 $[M–H]^-$, 243 $[M–COOH]^-$. $^1$H NMR (400 MHz, DMSO): δ = 3.64 (s, 2H, $CH_2$), 7.37–7.40 (m, 3H, ArH), 7.47–7.51 (m, 2H, ArH), 7.67–7.69 (m, 2H, ArH), 7.72–7.74 (m, 2H, ArH), 7.75–7.78 (m, 4H, ArH), 12.36 (brs, 1H, COOH) ppm. $^{13}$C NMR (100 MHz, DMSO) δ = 40.8 ($CH_2$), 126.9, 127.0, 127.52, 127.56, 127.7, 128.0, 129.5, 130.5, 134.9, 138.4, 139.4, 139.5, 140.1, 173.11 ppm.

## 4. References


[1] Watanabe, Y.; Miyawaki, Y.; Adachi, S.; Adachi, S.; Nakanishi, K.; Matsuno, R. Continuous production of acyl mannoses by immobilized lipase using a packed-bed reactor and their surfactant properties. *Biochem. Eng. J.* **2001**, *8,* 213–216.

[2] AlFindee, M.N.; Zhang, Q.; Subedi, Y.P.; Shrestha, J.P.; Kawasaki, Y.; Grilley, M.; Takemoto, J.Y.; Chang, C.W.T. One-step synthesis of carbohydrate esters as antibacterial and antifungal agents. *Bioorg. Med. Chem.* **2018**, *26*, 765–774.

[3] Yosimoto, K.; Tahara, K.; Suzuki, S.; Sasaki, K.; Nishikawa, Y.; Tsuda, Y. Regioselective syntheses of mono-*O*-acylglucoses. *Chem. Pharm. Bull.* **1979**, *27,* 2661–2674.

[4] Seath, C.P.; Fyfe, J.W.B.; Molloy, J.J.; Watson, A.J.B. Tandem chemoselective Suzuki-Miyaura cross-coupling enabled by nucleophile speciation control. *Angew. Chemie* **2015**, *54*, 9976–9979.

[5] Liphardt, B.; Luettke, W. laser dyes. I. Bifluorophoric laser dyes for increase of the efficient of dye lasers. *Liebigs Ann. Chem.* **1981**, *6*, 1118–1138.
